# Supplementary material for: Multi-context modeling of driver pathways reveals common and specific mechanisms across 23 cancer types
Source: PLoS Comput Biol. 2025 Aug 6;21(8):e1013349. doi: 10.1371/journal.pcbi.1013349 (PMC12349879; doi:10.1371/journal.pcbi.1013349)
Supplement: S1 Text — (PDF) [file pcbi.1013349.s001.pdf]

S1 Text: Supporting Information for "Multi-context modeling of driver pathways reveals common and specific mechanisms across 23 cancer types"

Wenjia Zhou, Junhua Zhang

This PDF file includes:
Section A to D
References

A. Hypergeometric test

For common genes shared by some cohorts, we examine the overlap between the cohorts' common IntOGen genes and their common driver genes discovered by EntCDP. Similarly, we examine the overlap between IntOGen genes unique to a specific cohort or several cohorts and the specific driver genes identified by ModSDP for that cohort or those cohorts relative to others. The significance of the overlap is determined using a hypergeometric test, and the overlaps are highlighted in dark colors in the Venn diagrams shown in S1 Fig.

B. Supplement to region-specific signaling pathways

Some cohorts of certain types of cancer lack driver gene information from IntOGen. In some cases, common IntOGen genes are only provided for part of, or a single, cohort, which reduces the accuracy of the test. However, it does not influence the identification of common gene sets by EntCDP (Table Cancers labeled # in S2 Table). For example, although IntOGen drivers of LUAD_ONCOSG are not provided, gene sets identified by EntCDP for cohort LUAD and LUAD_ONCOSG are significantly consistent with IntOGen genes of the cohort LUAD ($p < 0.001$). In particular, *EGFR*, *KRAS*, *BRAF*, *TP53*, *MAP2K1*, *RBM10* and *SMARCA4* are well-known driver genes in lung cancer [1, 2], while *NF1* and *CDKN2A* are less frequently reported but suggested as potential molecular targets [3, 4] (Table LUAD# in S2 Table). Similarly, four IntOGen genes, *IDH1*, *LRP1B*, *PIK3CA* and *PTEN*, in cohort GBM are common genes of cohorts GBM and GBM_DE (Table GBM# in S2 Table and Table S3.1 in S1 Data). It should be emphasized that the genes identified by EntCDP, including *CASP8*, *PIK3CA*, *MAP2K7*, and *PTEN*, may provide evidence for the involvement of the neurodegeneration pathway in the tumorigenesis of glioblastoma.

C. Supplement to pathway analysis of the tissues with similar location or related functions

Critical pathway acting in colorectal liver metastasis

It has been extensively reported that liver metastasis is the leading cause of cancer-related deaths in colorectal cancer [5, 6]. However, factors influencing the mutant patterns of metastases remain poorly understood.

In our study, we used EntCDP to identify the Wnt signaling pathway (represented by *CCND2*, *AXIN1*, *CTNNB1* and *TP53*; $K = 7$) as a common pathway for liver and colorectal cancers (S2A top and S2B Fig), which provides insight into the underlying mechanisms of cancer metastasis. *TP53* is both EntCDP and IntOGen gene of the two cohorts, while *AXIN1*, *CTNNB1* and *BAP1*, IntOGen genes of LIHC but not COADREAD, are reported as key defects of gastrointestinal cancers [7–9]. The remaining common genes, including *CCND2*, *CCND3* and *PPP2R1A*, are not found by IntOGen but all supported by the literature. Among them, splicing site mutations in *PPP2R1A* have been shown to be specific to liver metastatic cancer [10].

As for specific gene sets, ModSDP detected {*APC*, *BMPR1A*, *CCND3*, *ATG7*, *DAXX*, *DGCR8*} as COADREAD's specific pathway relative to LIHC. The first three genes are part of the Hippo signaling pathway (S2A bottom and S2B Fig), which plays a crucial role in maintaining intestinal homeostasis, and its dysregulation can lead to the occurrence and recurrence of colorectal cancer [11].

Two typical male urological tumors: KIRC and PRAD

Kidney and prostate are important for males, exerting essential functions in male fertility [12].

No common pathway genes for KIRC and PRAD were detected by EntCDP, which is consistent with the fact that the number of their common IntOGen genes is only three. With regard to specific identification, *STAG2* and *VHL* are significant ModSDP and IntOGen genes of KIRC, while for PRAD, the significant genes are *CDKN1B*, *FOXA1*, *HRAS*, *KDM6A*, and *SPOP*. Two gene sets {*FLCN*, *NKTR*, *RARA*, *UBR5*} and {*CAMTA1*, *PREX2*, *PTPRC*, *SMARCA1*} are not supported by the literature for either tumor group, but may provide therapeutic guidance for kidney and prostate cancer, respectively. When $K = 6$, we detected {*BCORL1*, *HSP90AA1*, *RARA*, *STAG2*, *UBR5*, *VHL*} as a PRAD-specific pathway relative to KIRC (Table S2.10 in S1 Data), among which *HSP90AA1*, *RARA*, *VHL* are related to Pathways in cancer. Conversely, transcriptional misregulation in cancer is the specific pathway for PRAD relative to KIRC with *CDKN1B*, *TP53* and *KDM6A* acting in it.

D. Supplement to the analysis of environmental factors

Smoke

Continued smoking is highly associated with not only a higher incidence but also greater risk of tumor recurrence and progression of bladder carcinoma [13]. Among specific gene sets for smokers, *KRAS* and *RB1* are two genes that are enriched in Bladder cancer and PI3K-Akt-mTOR signaling pathway (S3B Fig and Table S4.3 in S1 Data). This is consistent with previous findings that the invasion of nicotine exposure increases tumor growth and induces acquired chemoresistance through activation of the PI3K-Akt-mTOR pathway in bladder cancer [13]. *PSIP1*, *TRIM24*, and *RASA1* are specific genes in non-smokers and are enriched in the HIV life cell cycle pathway (Table S4.3 in S1 Data), which is an unexpected finding warrants further verified and tested.

Alcohol

Alcohol is also a risk factor for pancreatic cancer, as alcohol abstinence can alter the progression of pancreatitis and reduce recurrence [14]. When $K = 8$, we detected {*CD79B*, *FHIT*, *FLT4*, *FOXA2*, *KRAS*, *MEN1*, *MYO5A*, *PTEN*} in smokers, among

which *FLT4*, *PTEN* and *KRAS* are parts of the PI3K-Akt signaling pathway (S3D Fig and Table S4.6 in S1 Data), which has been reported as a target for effective therapy in pancreatic cancer [15]. Unfortunately, the results for non-smokers were not statistically significant.

References

1. Seo JS, Ju YS, Lee WC, Shin JY, Lee JK, Bleazard T, et al. The transcriptional landscape and mutational profile of lung adenocarcinoma. Genome research. 2012;22(11):2109–2119.

2. Chen H, Carrot-Zhang J, Zhao Y, Hu H, Freeman SS, Yu S, et al. Genomic and immune profiling of pre-invasive lung adenocarcinoma. Nature communications. 2019;10(1):1–6.

3. Wen S, Dai L, Wang L, Wang W, Wu D, Wang K, et al. Genomic signature of driver genes identified by target next-generation sequencing in Chinese non-small cell lung cancer. The oncologist. 2019;24(11):e1070.

4. Ding L, Getz G, Wheeler DA, Mardis ER, McLellan MD, Cibulskis K, et al. Somatic mutations affect key pathways in lung adenocarcinoma. Nature. 2008;455(7216):1069–1075.

5. Engstrand J, Nilsson H, Strömberg C, Jonas E, Freedman J. Colorectal cancer liver metastases–a population-based study on incidence, management and survival. BMC cancer. 2018;18(1):1–11.

6. Wang D, Liu J, Huo T, Tian Y, Zhao L. The role of microRNAs in colorectal liver metastasis: Important participants and potential clinical significances. Tumor Biology. 2017;39(6):1010428317709640.

7. Mazzoni SM, Fearon ER. AXIN1 and AXIN2 variants in gastrointestinal cancers. Cancer letters. 2014;355(1):1–8.

8. Tang J, Xi S, Wang G, Wang B, Yan S, Wu Y, et al. Prognostic significance of BRCA1-associated protein 1 in colorectal cancer. Medical Oncology. 2013;30(2):541.

9. Wu H, Lu XX, Wang JR, Yang TY, Li XM, He XS, et al. TRAF6 inhibits colorectal cancer metastasis through regulating selective autophagic CTNNB1/β-catenin degradation and is targeted for GSK3B/GSK3β-mediated phosphorylation and degradation. Autophagy. 2019;15(9):1506–1522.

10. Lu YW, Zhang HF, Liang R, Xie ZR, Luo HY, Zeng YJ, et al. Colorectal cancer genetic heterogeneity delineated by multi-region sequencing. PloS one. 2016;11(3):e0152673.

11. Huang Y, Yang F, Zhou T, Xie S. Emerging roles of Hippo signaling pathway in gastrointestinal cancers and its molecular mechanisms. Journal of Zhejiang University (Medical Science). 2020;49(1):35–43.

12. Motrich RD, Salazar FC, Breser ML, Mackern-Oberti JP, Godoy GJ, Olivera C, et al. Implications of prostate inflammation on male fertility. Andrologia. 2018;50(11):e13093.

13. Yuge K, Kikuchi E, Hagiwara M, Yasumizu Y, Tanaka N, Kosaka T, et al. Nicotine induces tumor growth and chemoresistance through activation of the PI3K/Akt/mTOR pathway in bladder cancer. Molecular cancer therapeutics. 2015;14(9):2112–2120.

14. Yadav D, Lowenfels AB. The epidemiology of pancreatitis and pancreatic cancer. Gastroenterology. 2013;144(6):1252–1261.

15. Ebrahimi S, Hosseini M, Shahidsales S, Maftouh M, A Ferns G, Ghayour-Mobarhan M, et al. Targeting the Akt/PI3K signaling pathway as a potential therapeutic strategy for the treatment of pancreatic cancer. Current medicinal chemistry. 2017;24(13):1321–1331.
