# Supplementary figures and images for: Multi-context modeling of driver pathways reveals common and specific mechanisms across 23 cancer types
Source: PLoS Comput Biol. 2025 Aug 6;21(8):e1013349. doi: 10.1371/journal.pcbi.1013349 (PMC12349879; doi:10.1371/journal.pcbi.1013349)

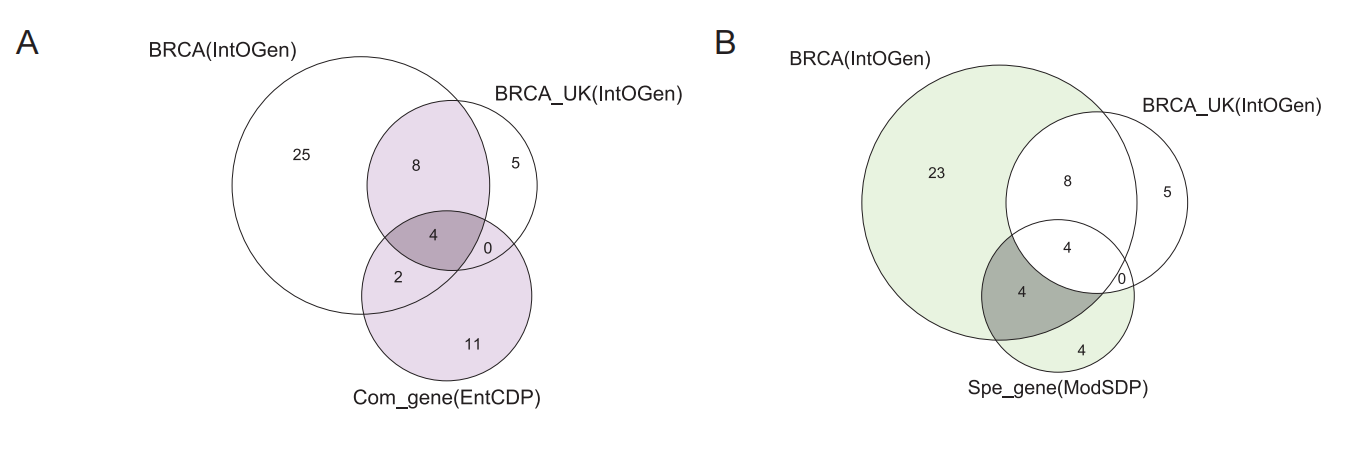

Supplement: S1 Fig — (TIF) [file pcbi.1013349.s002.tif]

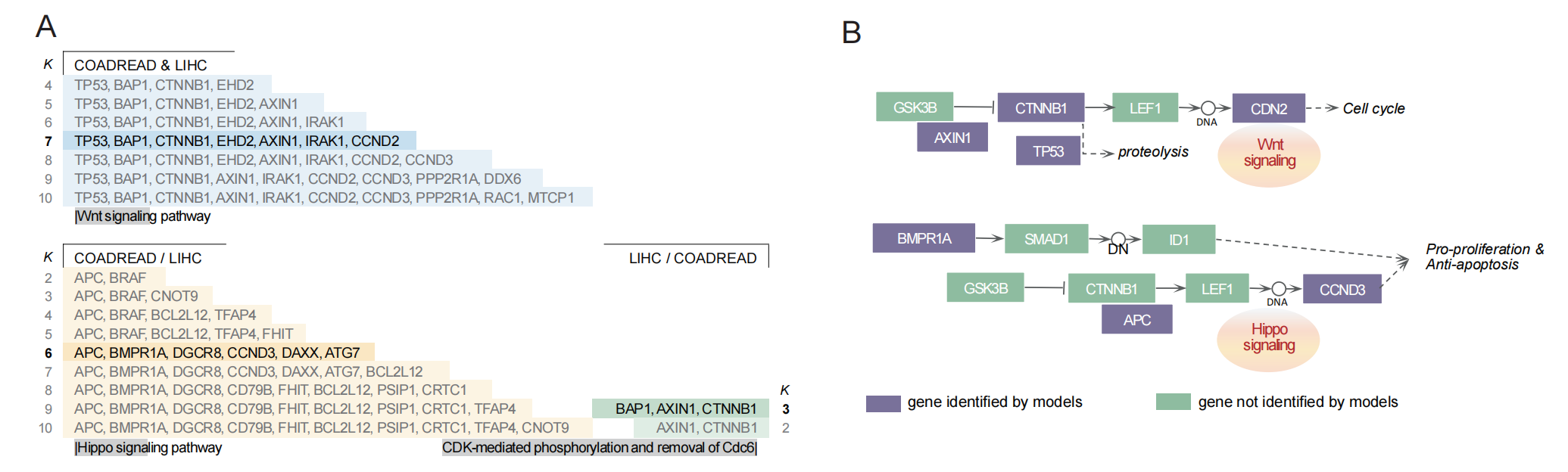

Supplement: S2 Fig — (TIF) [file pcbi.1013349.s003.tif]

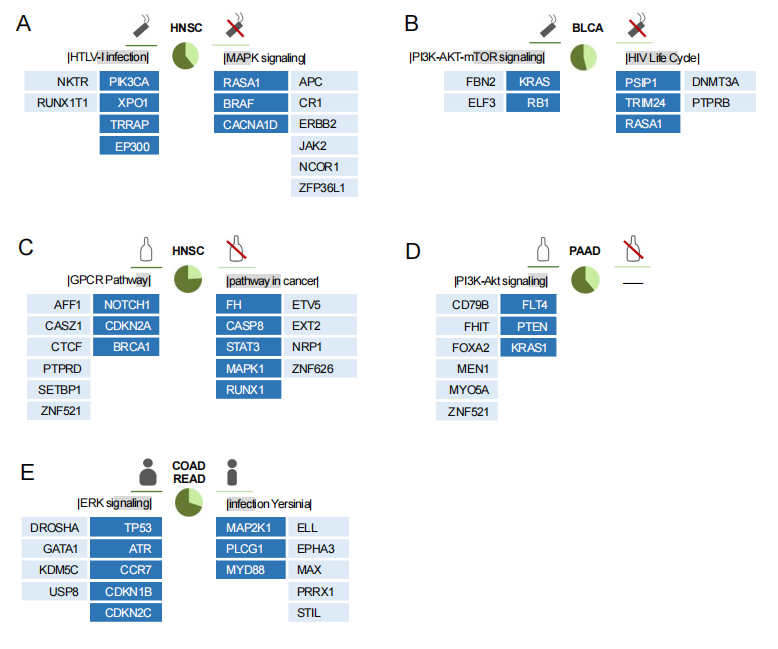

Supplement: S3 Fig — (TIF) [file pcbi.1013349.s004.tif]
