# Supplementary material for: Multi-context modeling of driver pathways reveals common and specific mechanisms across 23 cancer types
Source: PLoS Comput Biol. 2025 Aug 6;21(8):e1013349. doi: 10.1371/journal.pcbi.1013349 (PMC12349879; doi:10.1371/journal.pcbi.1013349)
Supplement: S1 Data — (PDF) [file pcbi.1013349.s008.pdf]

P.S.: p_i is the p-values of the individual significance of each identified gene sets for i^{th} cancer, p represents the p-value of overall significance.
Com2 (or 3): applying EntCDP to 2 (or 3) types of cancers. Spe: applying ModSDP to selected cancers.

1 Tables: Targeting novel driver genes and regionally specific signaling pathways

Bladder cancer

Com2

Table S1.1: Significant common driver gene set between BLCA and BLCA_CN

K	**common gene set**	p_1	p_2	p
3	*TP53,FGFR3,HRAS*	<0.0001	0.001	<0.0001
4	*TP53,FGFR3,HRAS,KRAS*	<0.0001	<0.0001	<0.0001
5	*TP53,FGFR3,HRAS,KRAS,FGFR2*	<0.0001	<0.0001	<0.0001
6	*TP53,FGFR3,HRAS,KRAS,FGFR2,PIK3CB*	<0.0001	0.001	<0.0001
7	*TP53,FGFR3,HRAS,KRAS,FGFR2,PIK3CB,CRTC1*	<0.0001	0.004	<0.0001
8	*TP53,FGFR3,HRAS,KRAS,FGFR2,PIK3CB,CRTC1,MB21D2*	<0.0001	<0.0001	<0.0001
9	*TP53,FGFR3,HRAS,KRAS,FGFR2,PIK3CB,CRTC1,MB21D2,USP44*	<0.0001	<0.0001	<0.0001
10	*TP53,FGFR3,HRAS,KRAS,FGFR2,PIK3CB,CRTC1,MB21D2,USP44,IDH2*	<0.0001	0.001	<0.0001

Spe

Table S1.2: BLCA and BLCA_CN specific mutated driver gene sets relative to each other

**Type**	K	**Specific gene set**	p	q	P
**BLCA / BLCA_CN**	2	*TP53,FGFR3*	<0.0001	0.25	<0.0001
	3	*KDM6A,KMT2D,RB1*	<0.0001	0.391	0.001
	4	*KDM6A,KMT2D,RB1,HOXC13*	<0.0001	0.407	<0.0001
	5	*KDM6A,KMT2D,RB1,HOXC13,IRS4*	<0.0001	0.408	<0.0001
	6	*KDM6A,KMT2D,PABPC1,IRS4,TRIM49C,CDKN1A*	<0.0001	0.775	<0.0001
	7	*KDM6A,KMT2D,PABPC1,IRS4,TRIM49C,HOXC13,ZNF93*	<0.0001	0.768	<0.0001
	8	*KDM6A,KMT2D,PABPC1,IRS4,TRIM49C,HOXC13,ZNF93,SMARCD1*	<0.0001	0.751	<0.0001
	9	*KDM6A,KMT2D,PABPC1,IRS4,TRIM49C,HOXC13,ZNF93,SMARCD1,IRAK1*	<0.0001	0.944	<0.0001
	10	*KDM6A,KMT2D,PABPC1,IRS4,TRIM49C,HOXC13,ZNF93,SMARCD1,IRAK1,CBL*	<0.0001	0.986	<0.0001
**BLCA_CN / BLCA**	10	*ARAF,BCL6,ESRRA,HLA-A,HLA-B,NTRK1,P2RY8,PTMA,RBFOX2,RGL3*	0.037	1	0.002

Breast adenocarcinoma

Com3

Table S1.3: Significant common driver gene set among BRCA, BRCA_EU and BRCA_UK

K	**common gene set**	p_1	p_2	p_3	p
3	*TP53,PIK3CA,GATA3*	<0.0001	0.001	0.002	<0.0001
4	*TP53,PIK3CA,GATA3,SEPT9*	<0.0001	0.005	0.004	<0.0001
5	*TP53,PIK3CA,GATA3,SEPT9,TAF15*	<0.0001	0.003	0.003	<0.0001
6	*TP53,PIK3CA,GATA3,SEPT9,TAF15,HIST1H4I*	<0.0001	0.001	0.006	<0.0001
7	*TP53,PIK3CA,GATA3,SEPT9,TAF15,HIST1H4I,H3F3A*	<0.0001	0.002	0.002	<0.0001
8	*TP53,PIK3CA,GATA3,SEPT9,TAF15,HIST1H4I,H3F3A,CARS*	<0.0001	<0.0001	0.007	<0.0001
9	*TP53,PIK3CA,GATA3,SEPT9,TAF15,HIST1H4I,H3F3A,CARS,FAM46C*	<0.0001	0.001	0.007	<0.0001
10	*TP53,PIK3CA,GATA3,SEPT9,TAF15,HIST1H4I,H3F3A,CARS,FAM46C,GTF2I*	<0.0001	0.003	0.006	<0.0001

Com2

Table S1.4: Significant common driver gene set between BRCA and BRCA_EU

K	**common gene set**	p_1	p_2	p
2	*TP53,PIK3CA*	<0.0001	0.01	<0.0001
3	*TP53,PIK3CA,GATA3*	<0.0001	<0.0001	<0.0001
4	*TP53,PIK3CA,GATA3,SEPT9*	<0.0001	<0.0001	<0.0001
5	*TP53,PIK3CA,GATA3,SEPT9,TAF15*	<0.0001	<0.0001	<0.0001
6	*TP53,PIK3CA,GATA3,SEPT9,TAF15,HIST1H4I*	<0.0001	0.002	<0.0001
7	*TP53,PIK3CA,GATA3,SEPT9,TAF15,HIST1H4I,H3F3A*	<0.0001	0.001	<0.0001
8	*TP53,PIK3CA,GATA3,SEPT9,TAF15,HIST1H4I,H3F3A,CARS*	<0.0001	<0.0001	0.007
9	*TP53,PIK3CA,GATA3,SEPT9,TAF15,HIST1H4I,H3F3A,CARS,FAM46C*	<0.0001	0.002	0.007
10	*TP53,PIK3CA,GATA3,SEPT9,TAF15,HIST1H4I,H3F3A,CARS,FAM46C,GTF2I*	<0.0001	0.001	0.006

Table S1.5: Significant common driver gene set between BRCA and BRCA_UK

K	**common gene set**	p_1	p_2	p
7	*TP53,GATA3,CDH1,CYSLTR2,MAP3K1,CDKN1B,IDH2*	<0.0001	0.041	<0.0001
8	*TP53,GATA3,CDH1,CYSLTR2,MAP3K1,SLC45A3,GNAI2,USP44*	<0.0001	0.032	<0.0001
9	*TP53,GATA3,CDKN1A,GNAI2,MYCN,PIK3CA,RECQL4,SEPT9,SIX1*	<0.0001	0.018	<0.0001
10	*TP53,GATA3,CDKN1A,GNAI2,MYCN,PIK3CA,RECQL4,SEPT9,SIX1,CDK4*	<0.0001	0.033	<0.0001

There is no common driver gene set between BRCA_EU and BRCA_UK when $K = 2 \sim 10$.

Spe

Table S1.6: BRCA specific mutated driver gene sets relative to BRCA_EU, UK.

K	**specific gene set**	p_1	p_2, p_3	p
3	*CDH1,MAP3K1,PIK3R1*	0.039	0.999,0.465	0.002
4	*CDH1,MAP2K4,PIK3R1,MAP3K1*	0.023	1,0.739	<0.0001
5	*CDH1,MAP2K4,PIK3R1,MAP3K1,BRCA1*	0.016	1,0.995	<0.0001
6	*CDH1,MAP2K4,PIK3R1,MAP3K1,HIST1H3B,KMT2D*	0.013	1,0.835	<0.0001
7	*CDH1,MAP2K4,PIK3R1,MAP3K1,BRCA1,HIST1H3B,CDKN1B*	0.007	1,0.991	<0.0001
8	*CDH1,MAP2K4,PIK3R1,MAP3K1,BRCA1,HIST1H3B,CDKN1B,PRDM1*	0.007	1,0.996	<0.0001
9	*CDH1,MAP2K4,PIK3R1,MAP3K1,JAK3,HIST1H3B,CDKN1B,PRDM1,KMT2D*	0.001	1,0.999	<0.0001
10	*CDH1,MAP2K4,PIK3R1,MAP3K1,JAK3,HIST1H3B,CDKN1B,PRDM1,KMT2D,BRCA1*	0.007	1,1	<0.0001

BRCA_UK has no specific mutated driver gene sets relative to BRCA and BRCA_EU when $K = 2 \sim 10$.

There is no specific driver gene set between BRCA and BRCA_EU relative to each other when $K = 2 \sim 10$.

There is no specific driver gene set between BRCA_EU and BRCA_UK relative to each other when $K = 2 \sim 10$.

Table S1.7: BRCA specific mutated driver gene sets relative to BRCA_UK

K	**specific gene set**	p_1	p_2	p
4	*TP53,CDH1,GATA3,MAP3K1*	<0.0001	0.07	<0.0001
5	*TP53,CDH1,GATA3,MAP3K1,MAP2K4*	<0.0001	0.164	<0.0001
6	*TP53,CDH1,GATA3,MAP3K1,AFF3,CTCF*	<0.0001	0.974	<0.0001
7	*AKT1,PIK3R1,BCORL1,KDM5A,MED12,PIK3CA,PTEN*	<0.0001	0.993	<0.0001
8	*AKT1,PIK3R1,BCORL1,KDM5A,MED12,PIK3CA,PTEN,PLCG1*	<0.0001	0.999	<0.0001
9	*AKT1,PIK3R1,BRCA2,CDH1,KMT2D,MAP3K1,NCOR1,RB1,ATM*	0.006	1	0.002
10	*AKT1,PIK3R1,BRCA2,CDH1,KMT2D,MAP3K1,NCOR1,RB1,ATM,MLLT3*	0.008	1	<0.0001

BRCA_UK has no specific mutated driver gene sets relative to BRCA when $K = 2 \sim 10$.

Colorectal adenocarcinoma

Com2

Table S1.8: Significant common driver gene set between COADREAD and COCA_CN

K	**common gene set**	p_1	p_2	p
3	*TP53,HLA-A,POU2F2*	0.03	<0.0001	<0.0001
4	*TP53,HLA-A,POU2F2,PPP2R1A*	<0.0001	<0.0001	<0.0001
5	*TP53,HLA-A,POU2F2,PPP2R1A,H3F3A*	0.003	<0.0001	<0.0001
6	*TP53,HLA-A,IRAK1,PPP2R1A,H3F3A,ZNF165*	0.001	<0.0001	<0.0001
7	*TP53,HLA-A,IRAK1,PPP2R1A,H3F3A,ZNF165,CEBPA*	<0.0001	0.002	<0.0001
8	*TP53,HLA-A,IRAK1,PPP2R1A,H3F3A,ZNF165,HIST1H4I,CEBPA*	0.034	0.001	<0.0001
9	*TP53,HLA-A,IRAK1,PPP2R1A,H3F3A,ZNF165,HIST1H4I,GTF2I,RARA*	0.002	0.001	<0.0001
10	*TP53,HLA-A,IRAK1,PPP2R1A,H3F3A,ZNF165,HIST1H4I,GTF2I,RARA,CEBPA*	<0.0001	0.025	<0.0001

Spe

Table S1.9: COADREAD specific mutated driver gene sets relative to COCA_CN

K	**specific gene set**	p_1	p_2	p
2	*APC,RNF43*	<0.0001	0.955	<0.0001
3	*APC,RNF43,NSD2*	<0.0001	1	<0.0001
4	*APC,RNF43,NSD2,EXT2*	<0.0001	1	<0.0001
5	*APC,CDKN2A,DNMT3A,EPHA2,GNA11*	<0.0001	1	<0.0001
6	*APC,CDKN2A,DNMT3A,EPHA2,GNA11,NSD2*	<0.0001	1	<0.0001
7	*APC,CCND3,BMPR1A,DAXX,DGCR8,NSD2,ATG7*	<0.0001	1	<0.0001
8	*APC,CCND3,BMPR1A,DAXX,DGCR8,NSD2,SH2B3,TFAP4*	<0.0001	1	<0.0001
9	*AJUBA,APC,BMPR1A,DAXX,DGCR8,NSD2,SH2B3,TFAP4,CCND3*	<0.0001	1	<0.0001
10	*AJUBA,APC,BMPR1A,DAXX,DGCR8,NSD2,SH2B3,TFAP4,CCND3,CDKN1A*	<0.0001	1	<0.0001

COCA_CN has no specific mutated driver gene sets relative to COADREAD when $K = 2 \sim 10$.

Esophageal cancer

Com3

There is no common driver gene set among ESCA, ESCA_CN and ESAD_UK when $K = 2 \sim 10$.

Com2

Table S1.10: Significant common driver gene set between ESCA and ESCA_CN

K	**common gene set**	p_1	p_2	p
3	*TP53,NEFH,PHF6*	0.006	<0.0001	<0.0001
4	*TP53,NEFH,PHF6,PSIP1*	0.006	<0.0001	<0.0001
5	*TP53,NEFH,PHF6,PSIP1,IDH2*	0.001	<0.0001	<0.0001
6	*TP53,BRD7,PHF6,PSIP1,RASA2,BIRC3*	0.033	<0.0001	<0.0001
7	*TP53,BRD7,PHF6,PSIP1,RASA2,BIRC3,IDH2*	0.016	<0.0001	<0.0001
8	*TP53,BRD7,PHF6,PSIP1,RASA2,BIRC3,IDH2,ELF4*	0.003	<0.0001	<0.0001
9	*TP53,BRD7,PHF6,PSIP1,RASA2,BIRC3,IDH2,ELF4,GNAI2*	<0.0001	<0.0001	<0.0001
10	*TP53,BRD7,PHF6,PSIP1,RASA2,BIRC3,IDH2,ELF4,GNAI2,SOX9*	<0.0001	<0.0001	<0.0001

There is no common driver gene set between ESCA and ESAD_UK when $K = 2 \sim 10$.

There is no common driver gene set between ESCA_CN and ESAD_UK when $K = 2 \sim 10$.

Spe

Table S1.11: ESCA specific mutated driver gene sets relative to ESCA_CN and ESAD_UK

K	**specific gene set**	p_1	p_2, p_3	p
2	*TP53,ZBTB20*	0.005	0.477,1	<0.0001
3	*TP53,ZBTB20,RBFOX1*	0.004	0.968,1	<0.0001
4	*TP53,ZBTB20,RBFOX1,FHIT*	0.013	0.913,1	<0.0001
5	*TP53,ZBTB20,RBFOX1,FHIT,EBF1*	0.001	1,0.097	<0.0001
7	*TP53,ZBTB20,RBFOX1,FHIT,EBF1,SFMBT2,AFF3*	0.003	0.998,1	<0.0001
8	*TP53,ZBTB20,RBFOX1,FHIT,EBF1,SFMBT2,AFF3,RUNX1*	0.011	0.999,1	<0.0001

Table S1.12: ESCA_CN specific mutated driver gene sets relative to ESCA and ESAD_UK

K	**specific gene set**	p_1	p_2, p_3	p
5	*TP53,EIF3E,FHIT,LPP,NTRK3*	0.027	0.299,1	0.005
8	*TP53,EIF3E,FHIT,LPP,NTRK3,RUNX1,SOHLH2,ALK*	0.032	0.642,1	0.011
9	*TP53,EIF3E,FHIT,LPP,NTRK3,RUNX1,SOHLH2,ALK,ESR1*	0.028	0.809,1	0.009

ESAD_UK has no specific mutated driver gene sets relative to ESCA and ESCA_CN when $K = 2 \sim 10$.

Table S1.13: ESCA specific mutated driver gene sets relative to ESAD_UK

K	**common gene set**	p_1	p_2	p
4	*TP53,FHIT,RBFOX1,ZBTB20*	0.012	1	<0.0001
5	*TP53,FHIT,RBFOX1,ZBTB20,ZNF521*	0.001	1	<0.0001
6	*TP53,FHIT,RBFOX1,ZBTB20,ZNF521,AFF3*	<0.0001	1	<0.0001
7	*TP53,FHIT,RBFOX1,ZBTB20,ZNF521,AFF3,EBF1*	0.001	1	<0.0001
8	*TP53,FHIT,RBFOX1,ZBTB20,ZNF521,AFF3,ALK,EBF1*	<0.0001	1	<0.0001
9	*TP53,FHIT,RBFOX1,ZBTB20,ZNF521,AFF3,ALK,EBF1,RUNX1*	0.005	1	<0.0001
10	*TP53,FHIT,RBFOX1,ZBTB20,ZNF521,AFF3,ALK,EBF1,RUNX1,SFMBT2*	0.009	1	0.002

ESAD_UK has no specific mutated driver gene sets relative to ESCA when $K = 2 \sim 10$.

Table S1.14: ESCA and ESCA_CN specific mutated driver gene sets relative to each other

**Type**	K	**Specific gene set**	p_1	p_2	p
**ESCA / ESCA_CN**	4	*GNAS,NFE2L2,APC,ARID1A*	0.012	0.913	0.005
	5	*GNAS,NFE2L2,NRG1,PIK3R1,SRGAP3*	<0.0001	0.972	<0.0001
	6	*GNAS,KLHL6,NRG1,PIK3R1,SRGAP3,NFE2L2*	<0.0001	0.961	<0.0001
	7	*GNAS,KLHL6,NRG1,PIK3R1,SRGAP3,NFE2L2,APC*	<0.0001	0.884	<0.0001
	8	*GNAS,KLHL6,NRG1,PIK3R1,SRGAP3,NFE2L2,APC,MAP3K1*	<0.0001	0.943	<0.0001
	9	*GNAS,KLHL6,BMPR1A,DCAF12L2,NFE2L2,APC,MAP3K1,SMAD4,CCND2*	<0.0001	0.875	<0.0001
	10	*GNAS,KLHL6,BMPR1A,DCAF12L2,NFE2L2,APC,MAP3K1,SMAD4,CCND2,SET*	<0.0001	0.972	<0.0001
**ESCA_CN / ESCA**	4	*FN1,PDE4DIP,PTPRB,NOTCH1*	<0.0001	0.536	0.005
	5	*FN1,PDE4DIP,PTPRB,NOTCH1,SP140*	0.001	0.537	0.002
	6	*FN1,PDE4DIP,PTPRB,NOTCH1,SP140,NEFH*	0.004	0.425	0.001
	7	*FN1,PDE4DIP,PTPRB,NOTCH1,SP140,NEFH,FANCD2*	<0.0001	0.9	0.002
	8	*FN1,PDE4DIP,PTPRB,NOTCH1,SP140,NEFH,FANCD2,STAT6*	<0.0001	0.856	<0.0001
	9	*FN1,PDE4DIP,PTPRB,NEFH,FAT1,KDM6A,MYH11,HERC2,ZNF814*	<0.0001	0.719	0.001
	10	*FN1,PDE4DIP,PTPRB,NOTCH1,SP140,NEFH,NFKBIE,STAT6,EBF1,HTRA2*	<0.0001	0.955	<0.0001

Glioblastoma multiforme

Com2

Table S1.15: Significant common driver gene set between GBM and GBM_MSKCC

K	**common gene set**	p_1	p_2	p
4	*IDH1,PIK3CA,PIK3R1,PTEN*	0.003	<0.0001	<0.0001
5	*IDH1,PIK3CA,PIK3R1,PTEN,H3F3A*	<0.0001	<0.0001	<0.0001
6	*IDH1,PIK3CA,PIK3R1,PTEN,H3F3A,ZNF208*	<0.0001	<0.0001	<0.0001
7	*IDH1,PIK3CA,PIK3R1,PTEN,H3F3A,ZNF208,BRAF*	0.014	<0.0001	<0.0001
8	*IDH1,PIK3CA,PIK3R1,PTEN,H3F3A,ZNF208,BRAF,LPAR4*	<0.0001	<0.0001	<0.0001
9	*IDH1,PIK3CA,PIK3R1,PTEN,H3F3A,ZNF208,BRAF,LPAR4,SGK1*	<0.0001	<0.0001	<0.0001
10	*IDH1,PIK3CA,PIK3R1,PTEN,H3F3A,ZNF208,BRAF,LPAR4,SGK1,NUP214*	<0.0001	<0.0001	<0.0001

Spe

Table S1.16: GBM_MSKCC specific mutated driver gene sets relative to GBM

K	**specific gene set**	p_1	p_2	p
2	*TP53,EGFR*	<0.0001	0.135	<0.0001
3	*TP53,EGFR,NF1*	<0.0001	0.082	<0.0001
4	*TP53,EGFR,NF1,BRAF*	<0.0001	0.15	0.001
5	*BIRC3,BRAF,EGFR,NF1,PDGFRA*	<0.0001	0.438	0.002
6	*BIRC3,BRAF,EGFR,NF1,PDGFRA,SPOP*	<0.0001	0.442	<0.0001
7	*BIRC3,NRAS,EGFR,NF1,PDGFRA,SPOP,FOXP1*	<0.0001	0.547	<0.0001
8	*BIRC3,NRAS,EGFR,NF1,PDGFRA,SPOP,FOXP1,SOCS1*	<0.0001	0.533	<0.0001
9	*BIRC3,NRAS,EGFR,NF1,PDGFRA,SPOP,FOXP1,SOCS1,KAT6B*	<0.0001	0.83	<0.0001
10	*BIRC3,NRAS,EGFR,NF1,PDGFRA,SPOP,FOXP1,SOCS1,KAT6B,RALGDS*	<0.0001	0.957	<0.0001

GBM has no specific mutated driver gene sets relative to GBM_MSKCC when $K = 2 \sim 10$.

Acute myeloid leukemia

Com3

There is no common driver gene set among LAML, LAML_CN and LAML_KR when $K = 2 \sim 10$.

Com2

There is no common driver gene set between LAML and LAML_CN when $K = 2 \sim 10$.

There is no common driver gene set between LAML and LAML_KR when $K = 2 \sim 10$.

There is no common driver gene set between LAML_CN and LAML_KR when $K = 2 \sim 10$.

Spe

Table S1.17: LAML specific mutated driver gene sets relative to LAML_CN and LAML_KR

K	**specific gene set**	p_1	p_2, p_3	p
3	*TP53,NPM1,RUNX1*	0.007	1,1	<0.0001
4	*TP53,NPM1,RUNX1,KIT*	0.002	1,1	<0.0001
5	*TP53,NPM1,RUNX1,KIT,ARHGAP35*	<0.0001	1,1	<0.0001
6	*TP53,NPM1,RUNX1,KIT,ARHGAP35,ETV6*	<0.0001	1,1	<0.0001
7	*TP53,NPM1,RUNX1,KIT,ARHGAP35,ETV6,KRAS*	<0.0001	1,1	<0.0001
8	*TP53,NPM1,RUNX1,KIT,ARHGAP35,ETV6,KRAS,JAK3*	<0.0001	1,1	<0.0001
9	*TP53,NPM1,RUNX1,KIT,ARHGAP35,ETV6,KRAS,JAK3,EHD2*	<0.0001	1,1	<0.0001
10	*TP53,NPM1,RUNX1,KIT,ARHGAP35,ETV6,KRAS,JAK3,EHD2,TET1*	<0.0001	1,1	<0.0001

LAML_CN has no specific mutated driver gene sets relative to LAML and LAML_KR when $K = 2 \sim 10$.

LAML_KR has no specific mutated driver gene sets relative to LAML and LAML_CN when $K = 2 \sim 10$.

There is no specific driver gene set between LAML_CN and LAML_KR relative to each other when $K = 2 \sim 10$.

Table S1.18: LAML specific mutated driver gene sets relative to LAML_CN

K	**common gene set**	p_1	p_2	p
3	*TP53,NPM1,RUNX1*	0.011	1	0.008
4	*TP53,NPM1,RUNX1,KIT*	<0.0001	1	0.001
5	*TP53,NPM1,RUNX1,KIT,FLT3*	0.001	1	0.007
6	*TP53,NPM1,RUNX1,KIT,FLT3,WT1*	0.001	1	0.001
7	*TP53,NPM1,RUNX1,KIT,FLT3,WT1,ARHGAP35*	<0.0001	1	<0.0001
8	*TP53,NPM1,RUNX1,KIT,FLT3,WT1,ARHGAP35,KRAS*	<0.0001	1	0.001
9	*TP53,NPM1,RUNX1,KIT,FLT3,WT1,ARHGAP35,KRAS,SMC1A*	<0.0001	1	<0.0001
10	*TP53,NPM1,RUNX1,KIT,FLT3,WT1,ARHGAP35,KRAS,SMC1A,GATA2*	<0.0001	1	<0.0001

LAML_CN has no specific mutated driver gene sets relative to LAML when $K = 2 \sim 10$.

Table S1.19: LAML specific mutated driver gene sets relative to LAML_KR

K	**specific gene set**	p_1	p_2	p
3	*TP53,ASXL1,NPM1*	0.042	1	<0.0001
4	*TP53,ASXL1,NPM1,ARHGAP35*	0.009	1	<0.0001
5	*TP53,ASXL1,NPM1,ARHGAP35,CBFB*	0.011	1	<0.0001
6	*TP53,ASXL1,NPM1,ARHGAP35,CBFB,SRSF2*	0.001	1	<0.0001
7	*TP53,ASXL1,NPM1,ARHGAP35,CBFB,SRSF2,H3F3A*	0.003	1	<0.0001
8	*TP53,ASXL1,NPM1,ARHGAP35,CBFB,SRSF2,H3F3A,MYD88*	0.001	1	<0.0001
9	*TP53,ASXL1,NPM1,ARHGAP35,CBFB,SRSF2,H3F3A,MED12,BCORL1*	0.004	1	<0.0001
10	*TP53,ASXL1,NPM1,ARHGAP35,CBFB,SRSF2,H3F3A,MED12,BCORL1,QKI*	0.005	1	<0.0001

LAML_KR has no specific mutated driver gene sets relative to LAML when $K = 2 \sim 10$.

Liver hepatocellular carcinoma

Com4

There is no common driver gene set among LIHC, LICA_CN, LICA_FR and LINC_JP when $K = 2 \sim 10$.

Com3

There is no common driver gene set among LIHC, LICA_FR and LICA_CN when $K = 2 \sim 10$.

Table S1.20: Significant common driver gene set among LIHC, LICA_FR and LINC_JP

K	**common gene set**	p_1	p_2	p_3	p
2	*TP53,CTNNB1*	0.026	0.015	0.005	<0.0001
3	*TP53,CTNNB1,BAP1*	0.005	0.001	0.005	<0.0001
4	*TP53,CTNNB1,BAP1,CDKN1A*	0.006	0.001	0.009	<0.0001
5	*TP53,CTNNB1,BAP1,CDKN1A,HIST1H3B*	0.007	<0.0001	0.005	<0.0001
6	*TP53,CTNNB1,BAP1,CDKN1A,HIST1H3B,PCBP1*	0.004	<0.0001	0.011	<0.0001
7	*TP53,CTNNB1,BAP1,CDKN1A,HIST1H3B,PCBP1,TAF15*	0.002	<0.0001	0.015	<0.0001
8	*TP53,CTNNB1,BAP1,CDKN1A,HIST1H3B,PCBP1,TAF15,H3F3A*	0.002	0.001	0.009	<0.0001
9	*TP53,CTNNB1,BAP1,CDKN1A,HIST1H3B,PCBP1,TAF15,H3F3A,RPS3A*	<0.0001	<0.0001	0.025	<0.0001
10	*TP53,CTNNB1,BAP1,CDKN1A,HIST1H3B,PCBP1,TAF15,H3F3A,RPS3A,KLF5*	0.001	<0.0001	0.013	<0.0001

There is no common driver gene set between LIHC, LICA_CN and LINC_JP when $K = 2 \sim 10$.

There is no common driver gene set between LICA_CN, LICA_FR and LINC_JP when $K = 2 \sim 10$.

Com2

There is no common driver gene set between LIHC and LICA_CN when $K = 2 \sim 10$.

There is no common driver gene set between LICA_CN and LICA_FR when $K = 2 \sim 10$.

There is no common driver gene set between LICA_CN and LINC_JP when $K = 2 \sim 10$.

Table S1.21: Significant common driver gene set between LIHC and LICA_FR

K	**common gene set**	p_1	p_2	p
2	*TP53,CTNNB1*	0.015	0.011	<0.0001
3	*TP53,CTNNB1,BAP1*	<0.0001	0.007	<0.0001
4	*TP53,CTNNB1,BAP1,ZXDB*	<0.0001	0.003	<0.0001
5	*TP53,CTNNB1,BAP1,CDKN1A,HIST1H3B*	0.001	0.012	<0.0001
6	*TP53,CTNNB1,BAP1,CDKN1A,HIST1H3B,KLF5*	<0.0001	0.005	<0.0001
7	*TP53,CTNNB1,BAP1,CDKN1A,HIST1H3B,KLF5,MYCN*	<0.0001	0.011	<0.0001
8	*TP53,CTNNB1,BAP1,DCAF12L2,HIST1H3B,KLF5,SMARCD1,BCL10*	<0.0001	0.006	<0.0001
9	*TP53,CTNNB1,BAP1,DCAF12L2,HIST1H3B,KLF5,SMARCD1,BCL10,PTPN6*	<0.0001	0.001	<0.0001
10	*TP53,CTNNB1,BAP1,DCAF12L2,HIST1H3B,KLF5,SMARCD1,BCL10,PTPN6,TAF15*	<0.0001	0.004	<0.0001

Table S1.22: Significant common driver gene set between LIHC and LINC_JP

K	**common gene set**	p_1	p_2	p
2	*TP53,CTNNB1*	0.023	0.005	0.001
3	*TP53,CTNNB1,BAP1*	<0.0001	0.006	<0.0001
4	*TP53,CTNNB1,BAP1,CDKN1A*	<0.0001	0.001	<0.0001
5	*TP53,CTNNB1,BAP1,CDKN1A,PCBP1*	<0.0001	0.003	<0.0001
6	*TP53,CTNNB1,BAP1,CDKN1A,PCBP1,HIST1H3B*	<0.0001	0.002	<0.0001
7	*TP53,CTNNB1,BAP1,CDKN1A,PCBP1,HIST1H3B,SOCS1*	<0.0001	0.004	<0.0001
8	*TP53,CTNNB1,BAP1,CDKN1A,PCBP1,HIST1H3B,SOCS1,FOXL2*	<0.0001	0.002	<0.0001
9	*TP53,CTNNB1,BAP1,CDKN1A,PCBP1,HIST1H3B,SOCS1,FOXL2,AXIN1*	<0.0001	<0.0001	<0.0001
10	*TP53,CTNNB1,BAP1,CDKN1A,PCBP1,HIST1H3B,SOCS1,FOXL2,AXIN1,TAF15*	<0.0001	0.001	<0.0001

Table S1.23: Significant common driver gene set between LICA_FR and LINC_JP

K	**common gene set**	p_1	p_2	p
2	*TP53,CTNNB1*	0.009	0.004	<0.0001
3	*TP53,CTNNB1,ZXDB*	0.003	0.009	<0.0001
4	*TP53,CTNNB1,ZXDB,RPS3A*	0.01	0.003	<0.0001
5	*TP53,CTNNB1,ZXDB,RPS3A,CARS*	0.011	0.004	<0.0001
6	*TP53,CTNNB1,ZXDB,RPS3A,CARS,CYLD*	0.006	0.002	<0.0001
7	*TP53,CTNNB1,ZXDB,RPS3A,CARS,CYLD,PCBP1*	0.013	0.005	<0.0001
8	*TP53,CTNNB1,ZXDB,RPS3A,CARS,CYLD,PCBP1,CEBPA*	0.015	0.001	<0.0001
9	*TP53,CTNNB1,ZXDB,RPS3A,CARS,CYLD,PCBP1,CEBPA,IRAK1*	0.019	0.003	<0.0001
10	*TP53,CTNNB1,ZXDB,RPS3A,CARS,CYLD,PCBP1,CEBPA,IRAK1,SOX21*	0.019	0.003	<0.0001

Spe

LICA_CN has no specific mutated driver gene sets relative to LIHC, LICA_FR and LINC_JP when $K = 2 \sim 10$.

LICA_FR has no specific mutated driver gene sets relative to LIHC, LICA_CN and LINC_JP when $K = 2 \sim 10$.

LICA_FR has no specific mutated driver gene sets relative to LIHC and LICA_CN when $K = 2 \sim 10$.

LICA_FR has no specific mutated driver gene sets relative to LIHC and LINC_JP when $K = 2 \sim 10$.

LICA_FR has no specific mutated driver gene sets relative to LICA_CN and LINC_JP when $K = 2 \sim 10$.

LICA_CN has no specific mutated driver gene sets relative to LIHC and LICA_FR when $K = 2 \sim 10$.

LICA_CN has no specific mutated driver gene sets relative to LIHC and LINJ_JP when $K = 2 \sim 10$.

LICA_CN has no specific mutated driver gene sets relative to LICA_FR and LINC_JP when $K = 2 \sim 10$.

Table S1.24: LIHC specific mutated driver gene sets relative to LICA_CN, LICA_FR and LINC_JP

K	**specific gene set**	p_1	p_2, p_3, p_4	p
4	*TP53,ARID1A,BAP1,CTNNB1*	<0.0001	1,0.398,0.38	<0.0001
5	*TP53,ARID1A,BAP1,CTNNB1,KEL*	<0.0001	1,0.772,0.865	<0.0001
6	*TP53,BAP1,CDKN1B,DCSTAMP,NRAS,IRS4*	0.005	1,0.984,1	<0.0001
7	*TP53,BAP1,CDKN1B,DCSTAMP,NRAS,CDKN1A,DCAF12L2*	<0.0001	1,0.988,0.962	<0.0001
8	*TP53,BAP1,CDKN1B,DCSTAMP,NRAS,CDKN1A,DCAF12L2,PRF1*	<0.0001	1,0.995,0.966	<0.0001
9	*TP53,BAP1,CDKN1B,DCSTAMP,NRAS,CDKN1A,DCAF12L2,TAF1,HIST1H3B*	0.001	1,0.993,0.967	<0.0001
10	*TP53,BAP1,APC,BRD4,CCDC6,CCND2,FGD5,IRS4,MLLT1,TRIM49C*	0.003	1,1,1	<0.0001

Table S1.25: LINC_JP specific mutated driver gene sets relative to LIHC, LICA_CN and LICA_FR

K	**specific gene set**	p_1	p_2, p_3, p_4	p
3	*AR,CTNNB1,TOP2A*	0.027	0.565,1,0.924	<0.0001
4	*AR,CTNNB1,TOP2A,NEFH*	0.004	0.6224,1,0.994	<0.0001
5	*AR,CTNNB1,TOP2A,NEFH,CDKN1A*	<0.0001	0.617,1,0.999	<0.0001
6	*AR,CTNNB1,TOP2A,NEFH,CDKN1A,NPRL2*	0.002	0.613,1,0.998	<0.0001
7	*AR,CTNNB1,TOP2A,NEFH,CDKN1A,NPRL2,DNAJB1*	<0.0001	0.545,1,1	<0.0001
8	*AR,CTNNB1,TOP2A,NEFH,CDKN1A,NPRL2,DNAJB1,RBM38*	<0.0001	0.518,1,1	<0.0001
9	*AR,CTNNB1,TOP2A,NEFH,CDKN1A,NPRL2,DNAJB1,RBM38,LTB*	<0.0001	0.499,1,1	<0.0001
10	*AR,CTNNB1,TOP2A,NEFH,CDKN1A,NPRL2,DNAJB1,RBM38,LTB,UBE2A*	<0.0001	0.671,1,1	<0.0001

Table S1.26: LINC_JP specific mutated driver gene sets relative to LIHC and LICA_FR

K	**specific gene set**	p_1	p_2, p_3	p
8	*ALB,COL1A1,NEFH,RGL3,DCAF12L2,HLA-A,PRR14*	0.008	0.897,1	<0.0001
9	*ALB,COL1A1,NEFH,RGL3,HOXD13,TFG,CXCR4,SMARCB1,FOXD4L1*	0.002	0.901,1	<0.0001
10	*ALB,COL1A1,NEFH,RGL3,HOXD13,TFG,CXCR4,DCAF12L2,PPM1D,SOX17*	0.001	0.893,1	<0.0001

Table S1.27: LINC_JP specific mutated driver gene sets relative to LIHC and LICA_CN / LICA_CN and LICA_FR

K	**common gene set**	p_1	p_2, p_3	p
3	*AR,CTNNB1,TOP2A*	0.027	1,0.936	<0.0001
4	*FLCN,CTNNB1,TOP2A,NEFH*	0.009	1,1	<0.0001
5	*AR,CTNNB1,TOP2A,NEFH,CDKN1A*	<0.0001	1,1	<0.0001
6	*FLCN,CTNNB1,TOP2A,NEFH,CDKN1A,NPRL2*	0.001	1,1	<0.0001
7	*AR,CTNNB1,TOP2A,NEFH,CDKN1A,NPRL2,DNAJB1*	<0.0001	1,1	<0.0001
8	*AR,CTNNB1,TOP2A,NEFH,CDKN1A,NPRL2,DNAJB1,RBM38*	<0.0001	1,1	<0.0001
9	*AR,CTNNB1,TOP2A,NEFH,CDKN1A,NPRL2,DNAJB1,RBM38,LTB*	<0.0001	1,1	<0.0001
10	*AR,CTNNB1,TOP2A,NEFH,CDKN1A,NPRL2,DNAJB1,RBM38,LTB,UBE2A*	<0.0001	1,1	<0.0001

Table S1.28: LIHC specific mutated driver gene sets relative to LICA_CN

K	**specific gene set**	p_1	p_2	p
2	*TP53,CTNNB1*	0.02	0.94	0.003
3	*AXIN1,BAP1,CTNNB1*	<0.0001	1	<0.0001
4	*AXIN1,BAP1,CTNNB1,RECQL4*	<0.0001	1	<0.0001
5	*AXIN1,BAP1,CTNNB1,RECQL4,SH2B3*	<0.0001	1	<0.0001
6	*AXIN1,BAP1,CTNNB1,RECQL4,SH2B3,RELA*	<0.0001	1	<0.0001
7	*AXIN1,BAP1,CTNNB1,RECQL4,SH2B3,RELA,NFKB2*	<0.0001	1	<0.0001
8	*AXIN1,BAP1,CTNNB1,RECQL4,SH2B3,RELA,NFKB2,PCBP1*	<0.0001	1	<0.0001
9	*AXIN1,BAP1,CTNNB1,RECQL4,SH2B3,RELA,NFKB2,PCBP1,ZXDB*	<0.0001	1	<0.0001
10	*AXIN1,BAP1,CTNNB1,RECQL4,SH2B3,RELA,NFKB2,PCBP1,ZXDB,DCAF12L2*	<0.0001	1	<0.0001

LICA_CN has no specific mutated driver gene sets relative to LIHC when $K = 2 \sim 10$.

Table S1.29: LIHC specific mutated driver gene sets relative to LICA_FR

K	**specific gene set**	p_1	p_2	p
6	*ALB,BIRC6,EBF1,LRP1B,NBEA,PTPRT*	0.025	1	<0.0001
7	*ALB,BIRC6,EBF1,LRP1B,NBEA,PTPRT,RASA1*	0.003	1	<0.0001
8	*ALB,BIRC6,EBF1,LRP1B,NBEA,PTPRT,RASA1,RUNX1*	0.003	1	<0.0001
9	*ALB,BIRC6,EBF1,LRP1B,NBEA,PTPRT,RASA1,RUNX1,KDR*	<0.0001	1	<0.0001
10	*ALB,BIRC6,EBF1,LRP1B,NBEA,PTPRT,RASA1,RUNX1,KDR,RGS7*	<0.0001	1	<0.0001

LICA_FR has no specific mutated driver gene sets relative to LIHC when $K = 2 \sim 10$.

Table S1.30: LIHC and LINC_JP specific mutated driver gene sets relative to each other

**Type**	K	**Specific gene set**	p_1	p_2	p
**LIHC / LINC_JP**	10	*BRD4,CCDC6,CPEB3,CR1,FGD5,IL6ST,JAK1,MLLT3,TET2,TRIM49C*	0.004	1	<0.0001
**LINC_JP / LIHC**	5	*ALB,COL1A1,HLA-A,NEFH,PRR14*	0.027	0.989	0.013

There is no specific driver gene set between LICA_CN and LICA_FR relative to each other when $K = 2 \sim 10$.

Table S1.31: LINC_JP specific mutated driver gene sets relative to LICA_CN

K	**specific gene set**	p_1	p_2	p
2	*CTNNB1,TOP2A*	0.046	1	<0.0001
3	*CTNNB1,TOP2A,CDKN1A*	0.006	1	<0.0001
4	*CTNNB1,TOP2A,CDKN1A,KLF4*	0.004	1	<0.0001
5	*CTNNB1,TOP2A,CDKN1A,KLF4,FOXA2*	0.002	1	<0.0001
6	*CTNNB1,TOP2A,CDKN1A,DNAJB1,FLCN,NEFH*	<0.0001	1	<0.0001
7	*CTNNB1,TOP2A,CDKN1A,KLF4,FOXA2,DNAJB1,NXF1*	<0.0001	1	<0.0001
8	*CTNNB1,TOP2A,CDKN1A,KLF4,FOXA2,DNAJB1,NEFH,RBM38*	<0.0001	1	<0.0001
9	*CTNNB1,TOP2A,CDKN1A,KLF4,FOXA2,DNAJB1,NEFH,RBM38,NPRL2*	<0.0001	1	<0.0001
10	*CTNNB1,TOP2A,CDKN1A,KLF4,FOXA2,DNAJB1,NEFH,RBM38,NPRL2,LTB*	<0.0001	1	<0.0001

LICA_CN has no specific mutated driver gene sets relative to LINC_JP when $K = 2 \sim 10$.

Table S1.32: LINC_JP specific mutated driver gene sets relative to LICA_FR

K	**specific gene set**	p_1	p_2	p
6	*ALB,DCAF12L2,COL1A1,HLA-A,NEFH,RGL3*	0.018	1	<0.0001
7	*ALB,DCAF12L2,COL1A1,HLA-A,NEFH,PRR14,RGL3*	0.006	1	<0.0001
8	*ALB,DCAF12L2,COL1A1,HLA-A,NEFH,PRR14,RGL3,BTG1*	0.008	1	<0.0001
9	*ALB,DCAF12L2,COL1A1,HLA-A,NEFH,PRR14,RGL3,BTG1,PRF1*	0.002	1	<0.0001
10	*ALB,DCAF12L2,HOXD13,KLHL36,NEFH,PPM1D,SF3B1,SH2B3,SOX17,TFG*	0.007	1	<0.0001

LICA_FR has no specific mutated driver gene sets relative to LINC_JP when $K = 2 \sim 10$.

Lung adenocarcinoma

Com3

Table S1.33: Significant common driver gene set among LUAD, LUAD_MSKCC and LUAD_ONCOSG

K	**common gene set**	p_1	p_2	p_3	p
2	*KRAS,EGFR*	<0.0001	<0.0001	<0.0001	<0.0001
3	*KRAS,EGFR,LRP1B*	<0.0001	<0.0001	<0.0001	<0.0001
4	*KRAS,EGFR,LRP1B,MET*	<0.0001	<0.0001	<0.0001	<0.0001
5	*KRAS,EGFR,BRAF,MET,NF1*	<0.0001	<0.0001	<0.0001	<0.0001
6	*KRAS,EGFR,BRAF,MET,NF1,ERBB2*	<0.0001	<0.0001	<0.0001	<0.0001
7	*KRAS,EGFR,BRAF,MET,NF1,ERBB2,BCLAF1*	<0.0001	<0.0001	<0.0001	<0.0001
8	*KRAS,EGFR,BRAF,MET,NF1,ERBB2,BCLAF1,NEFH*	<0.0001	<0.0001	<0.0001	<0.0001
9	*KRAS,EGFR,BRAF,MET,NF1,ERBB2,BCLAF1,NEFH,MAP2K1*	<0.0001	<0.0001	<0.0001	<0.0001
10	*KRAS,EGFR,BRAF,MET,NF1,ERBB2,BCLAF1,NEFH,MAP2K1,WNK4*	<0.0001	<0.0001	<0.0001	<0.0001

Com2

Table S1.34: Significant common driver gene set between LUAD and LUAD_MSKCC

K	**common gene set**	p_1	p_2	p
2	*TP53,KRAS*	<0.0001	0.001	<0.0001
3	*EGFR,KRAS,LRP1B*	0.003	<0.0001	<0.0001
4	*EGFR,KRAS,LRP1B,MET*	<0.0001	<0.0001	<0.0001
5	*EGFR,KRAS,LRP1B,MET,ERBB2*	<0.0001	<0.0001	<0.0001
6	*EGFR,KRAS,LRP1B,MET,ERBB2,BRAF*	<0.0001	<0.0001	<0.0001
7	*EGFR,KRAS,LRP1B,MET,ERBB2,BRAF,NF1*	<0.0001	<0.0001	<0.0001
8	*EGFR,KRAS,LRP1B,MET,ERBB2,BRAF,NEFH,KAT6A*	<0.0001	<0.0001	<0.0001
9	*EGFR,KRAS,BRAF,EIF3E,ERBB2,MET,NF1,RNF213,SMARCA1*	<0.0001	<0.0001	<0.0001
10	*EGFR,KRAS,BRAF,EIF3E,ERBB2,MET,NF1,CAMTA1,CHD2,WNK4*	<0.0001	<0.0001	<0.0001

Table S1.35: Significant common driver gene set between LUAD and LUAD_ONCOSG

K	**common gene set**	p_1	p_2	p
2	*TP53,RBM10*	0.031	0.012	0.001
8	*BRAF,EGFR,EPS15,KRAS,MET,NF1,NEFH,SMARCA4*	<0.0001	<0.0001	<0.0001
9	*BRAF,EGFR,EPS15,KRAS,MET,NF1,NEFH,SMARCA4,MAP2K1*	<0.0001	<0.0001	<0.0001
10	*BRAF,EGFR,ERBB2,KRAS,MET,NF1,SIN3A,ZNF626,MAP2K1,CDKN2A*	<0.0001	<0.0001	<0.0001

Table S1.36: Significant common driver gene set between LUAD_MSKCC and LUAD_ONCOSG

K	**common gene set**	p_1	p_2	p
2	*EGFR,KRAS*	<0.0001	<0.0001	<0.0001
3	*EGFR,KRAS,BRAF*	<0.0001	<0.0001	<0.0001
4	*EGFR,KRAS,BRAF,ERBB2*	<0.0001	<0.0001	<0.0001
5	*EGFR,KRAS,BRAF,ERBB2,MET*	<0.0001	<0.0001	<0.0001
6	*EGFR,KRAS,BRAF,ERBB2,MET,DHX9*	<0.0001	<0.0001	<0.0001
7	*EGFR,KRAS,BRAF,ERBB2,MET,DHX9,PARP4*	<0.0001	<0.0001	<0.0001
8	*EGFR,KRAS,BRAF,ERBB2,MET,DHX9,MAF,WRN*	<0.0001	<0.0001	<0.0001
9	*EGFR,KRAS,BRAF,ERBB2,MET,DHX9,MAF,MAP2K1,WRN*	<0.0001	<0.0001	<0.0001
10	*EGFR,KRAS,BRAF,ERBB2,MET,DHX9,MAF,MAP2K1,WRN,FANCC*	<0.0001	<0.0001	<0.0001

Spe

Table S1.37: LUAD specific mutated driver gene sets relative to LUAD_MSKCC and LUAD_ONCOSG

K	**specific gene set**	p_1	p_2, p_3	p
3	*TP53,ATM,DOT1L*	<0.0001	0.261,0.838	0.003
4	*TP53,ATM,DOT1L,ZFX*	<0.0001	0.222,0.942	<0.0001
8	*EWSR1,LRP1B,NBEA,NRK,PPP3CA,RRAGC,SFMBT2,ZNF780A*	0.041	1,1	0.004
9	*EWSR1,LRP1B,NBEA,NRK,PPP3CA,RRAGC,SFMBT2,ZNF780A,ZFX*	0.024	1,1	0.001
10	*EWSR1,LRP1B,NBEA,NRK,PPP3CA,RRAGC,SFMBT2,ZNF780A,ZFX,CNOT3*	0.012	1,1	<0.0001

Table S1.38: LUAD_MSKCC specific mutated driver gene sets relative to LUAD and LUAD_ONCOSG

K	**specific gene set**	p_1	p_2, p_3	p
5	*MAX,NKX2-1,RBM10,STK11,U2AF1*	0.04	0.97,0.732	0.016
6	*MAX,NKX2-1,RBM10,STK11,U2AF1,ARAF*	0.021	0.991,0.898	0.007
7	*MAX,NKX2-1,RBM10,STK11,U2AF1,ARAF,SRSF2*	0.015	0.993,0.901	0.002
8	*MAX,NKX2-1,RBM10,SRSF2,U2AF1,ARAF,STK11,FUBP1*	0.003	0.99,0.792	<0.0001
9	*MAX,NKX2-1,RBM10,SRSF2,U2AF1,ARAF,STK11,FUBP1,MYD88*	0.001	0.964,0.802	0.001
10	*MAX,NKX2-1,RBM10,SRSF2,U2AF1,ARAF,STK11,FUBP1,MYD88,CSF3R*	0.001	0.942,0.813	<0.0001

Table S1.39: LUAD_ONCOSG specific mutated driver gene sets relative to LUAD and LUAD_MSKCC

K	**specific gene set**	p_1	p_2, p_3	p
7	*ACSL3,EFTUD2,HSPG2,MAF,RBFOX1,RGS7,RHPN2*	0.008	1,1	0.002
8	*ACSL3,EFTUD2,HSPG2,MAF,RBFOX1,RGS7,RHPN2,SET*	0.002	1,1	<0.0001
10	*ACSL3,EFTUD2,HSPG2,NT5C3A,PARP4,RXRA,ZNF721,BCL2L12,DDX3X,FAM186A*	0.048	0.999,1	0.007

Table S1.40: LUAD and LUAD_MSKCC specific mutated driver gene sets relative to each other

**Type**	K	**Specific gene set**	p_1	p_2	p
**LUAD / LUAD_MSKCC**	9	*EWSR1,LRP1B,NBEA,NIN,NPM1,NRK,PPP3CA,RRAGC,TOP2A*	0.021	0.999	0.015
	10	*EWSR1,LRP1B,NBEA,NIN,NPM1,NRK,PPP3CA,RRAGC,TOP2A,RPS3A*	0.009	0.998	0.007
**LUAD_MSKCC / LUAD**	3	*NKX2-1,RBM10,MET*	0.001	0.554	0.006
	5	*NKX2-1,RBM10,MAX,U2AF1,STK11*	0.035	0.977	0.015
	6	*NKX2-1,RBM10,MAX,U2AF1,ARAF,SPOP*	0.019	0.981	0.006
	7	*NKX2-1,RBM10,MAX,U2AF1,ARAF,SPOP,CDC73*	0.017	0.999	0.003
	8	*NKX2-1,RBM10,MAX,U2AF1,ARAF,SPOP,CDC73,AR*	0.015	1	0.001
	9	*NKX2-1,RBM10,MAX,U2AF1,ARAF,SPOP,CDC73,AR,SH2B3*	0.003	0.999	<0.0001
	10	*NKX2-1,RBM10,MAX,U2AF1,ARAF,SPOP,CDC73,AR,SH2B3,SRSF2*	0.009	0.999	<0.0001

Table S1.41: LUAD and LUAD_ONCOSG specific mutated driver gene sets relative to each other

Type	K	Specific gene set	p_1	p_2	p
**LUAD / LUAD_ONCOSG**	3	*TP53,KRAS,MET*	<0.0001	0.967	<0.0001
	4	*TP53,KRAS,MET,ZFX*	<0.0001	0.983	<0.0001
	5	*TP53,KRAS,MET,ZFX,NPRL2*	<0.0001	0.994	<0.0001
	6	*TP53,KRAS,MET,ZFX,NPRL2,PTPN6*	<0.0001	0.999	<0.0001
	7	*TP53,KRAS,MET,ZFX,NPRL2,PTPN6,MAX*	<0.0001	0.995	<0.0001
	8	*TP53,ATM,DOT1L,HSP90AB1,MAX,MDM4,USP44,ZFX*	<0.0001	1	<0.0001
	9	*TP53,ATM,DOT1L,HSP90AB1,MAX,MDM4,USP44,ZFX,SOHLH2*	<0.0001	1	<0.0001
	10	*TP53,ATM,DOT1L,HSP90AB1,MAX,MDM4,USP44,ZFX,SOHLH2,SMC1A*	<0.0001	0.998	<0.0001
**LUAD_ONCOSG / LUAD**	2	*EGFR,SETD1B*	0.004	1	0.003
	3	*EGFR,SETD1B,PBRM1*	<0.0001	0.919	<0.0001
	4	*EGFR,SETD1B,PBRM1,CCND2*	<0.0001	0.957	<0.0001
	5	*EGFR,SETD1B,RBM38,HTRA2,IRF1*	<0.0001	0.873	<0.0001
	6	*EGFR,SETD1B,HTRA2,IRF1,MDM4,TGIF1*	<0.0001	0.875	<0.0001
	7	*EGFR,SETD1B,HTRA2,IRF1,MDM4,TGIF1,RBM38*	<0.0001	0.881	<0.0001

Table S1.42: LUAD_MSKCC and LUAD_ONCOSG specific mutated driver gene sets relative to each other

Type	K	Specific gene set	p_1	p_2	p
**LUAD_MSKCC / LUAD_ONCOSG**	3	*TP53,MET,RBM10*	0.004	0.235	<0.0001
	4	*TP53,MET,STK11,ATM*	0.002	0.578	0.001
	5	*TP53,MAX,STK11,ATM,NKX2-1*	0.003	0.638	0.002
	6	*TP53,MAX,STK11,ATM,NKX2-1,EIF1AX*	0.003	0.635	0.003
	7	*TP53,MAX,STK11,ATM,NKX2-1,EIF1AX,GNAS*	0.001	0.817	<0.0001
	8	*TP53,MAX,STK11,ATM,NKX2-1,EIF1AX,GNAS,SMAD3*	0.004	0.852	<0.0001
	9	*TP53,MAX,STK11,ATM,NKX2-1,EIF1AX,GNAS,SMAD3,GNA11*	0.001	0.859	<0.0001
	10	*TP53,MAX,STK11,ATM,NKX2-1,EIF1AX,GNAS,SMAD3,GNA11,PPP6C*	<0.0001	0.871	<0.0001
**LUAD_ONCOSG / LUAD_MSKCC**	7	*ARHGAP35,LRP1B,NEFH,NRK,RHPN2,RNF213,PRDM2*	0.017	1	0.012
	8	*ARHGAP35,LRP1B,NEFH,NRK,RHPN2,RNF213,SUSD2,COL1A1*	0.029	1	0.029
	9	*ARHGAP35,LRP1B,NEFH,NRK,RHPN2,RNF213,SUSD2,COL1A1,TNC*	0.017	1	0.022
	10	*ARHGAP35,LRP1B,NEFH,NRK,RHPN2,RNF213,SUSD2,COL1A1,TNC,PLAG1*	0.015	1	0.019

Lung squamous cell carcinoma

Com2

Table S1.43: Significant common driver gene set between LUSC and LUSC_KR

K	**common gene set**	p_1	p_2	p
5	*TP53,BAP1,PCMTD1,PIM1,SOCS1*	0.026	0.002	0.02
6	*TP53,BAP1,PCMTD1,PIM1,SOCS1,PCBP1*	0.009	0.017	<0.0001
7	*TP53,BAP1,PCMTD1,PIM1,SOCS1,PCBP1,MYD88*	<0.0001	0.028	<0.0001
8	*TP53,CCR7,KRAS,MYD88,NPRL2,PCBP1,RPL22,ZXDB*	0.01	0.003	<0.0001
9	*TP53,CCR7,KRAS,MYD88,NPRL2,PCBP1,RPL22,ZXDB,PRKCD*	0.002	0.006	<0.0001
10	*TP53,CCR7,KRAS,MYD88,NPRL2,PCBP1,RPL22,ZXDB,PRKCD,BAP1*	0.003	0.006	<0.0001

Spe

Table S1.44: LUSC and LUSC_KR specific mutated driver gene sets relative to each other

**Type**	K	**Specific gene set**	p_1	p_2	p
**LUSC / LUSC_KR**	5	*CUX1,FAM135B,FLT4,LRP1B,NFE2L2*	0.021	1	<0.0001
	6	*CUX1,FAM135B,FLT4,LRP1B,NFE2L2,RBFOX2*	0.01	1	<0.0001
	7	*CUX1,FLT4,LRP1B,NFE2L2,RB1,RGS7,AFF1*	<0.0001	1	<0.0001
	8	*ENPEP,FANCA,LRP1B,NFE2L2,PRDM2,RB1,SETD2,TLL1*	<0.0001	1	<0.0001
	9	*BTK,FN1,KEAP1,MAP2,NBEA,NFE2L2,PREX2,PTEN,PTPRC*	<0.0001	1	<0.0001
	10	*BTK,FN1,KEAP1,MAP2,NBEA,NFE2L2,PREX2,PTEN,PTPRC,MAP2K1*	<0.0001	1	<0.0001
**LUSC_KR / LUSC**	2	*LTB,PDE4DIP*	0.02	1	0.016
	3	*LTB,PDE4DIP,PCBP1*	0.026	1	0.002
	7	*LTB,PDE4DIP,SEPT9,FAM46C,H3F3A,HIST1H4I,KLF4*	0.049	0.842	0.044
	10	*LTB,HLA-B,SEPT9,RRAGC,SET,SETD1B,SLC45A3,SOCS1,TFAP4,UBE2A*	0.015	0.915	0.024

Ovarian cancer

Com2

There is no common driver gene set between OV and OV_AU when $K = 2 \sim 10$.

Spe

There is no specific driver gene set between OV and OV_AU relative to each other when $K = 2 \sim 10$.

Pancreatic adenocarcinoma

Com3

There is no common driver gene set among PAAD, PACA_AU and PACA_CA when $K = 2 \sim 10$.

Com2

There is no common driver gene set between PAAD and PACA_AU when $K = 2 \sim 10$.

There is no common driver gene set between PAAD and PACA_CA when $K = 2 \sim 10$.

There is no common driver gene set between PACA_AU and PACA_CA when $K = 2 \sim 10$.

Spe

Table S1.45: PACA_AU specific mutated driver gene sets relative to PAAD and PACA_CA

K	**specific gene set**	p_1	p_2, p_3	p
3	*BIRC3,CEBPA,KRAS*	0.043	0.448,1	0.011
4	*BIRC3,CEBPA,KRAS,FAM46C*	0.033	0.704,1	0.002
5	*BIRC3,CEBPA,KRAS,FAM46C,CARS*	0.031	0.688,1	0.008
6	*BIRC3,CEBPA,KRAS,FAM46C,CARS,TAF15*	0.021	0.601,1	0.008
7	*BIRC3,CEBPA,KRAS,FAM46C,CARS,TAF15,RPL10*	0.025	0.619,1	0.005
8	*BIRC3,CEBPA,KRAS,FAM46C,CARS,TAF15,RPL10,SOX17*	<0.0001	0.425,0.995	<0.0001
9	*BIRC3,CEBPA,KRAS,FAM46C,CARS,TAF15,RPL10,SOX17,MYCN*	0.002	0.668,0.945	<0.0001
10	*BIRC3,CEBPA,KRAS,FAM46C,CARS,TAF15,RPL10,SOX17,MYCN,EIF1AX*	0.006	0.691,0.989	<0.0001

PAAD has no specific mutated driver gene sets relative to PACA_AU and PACA_CA when $K = 2 \sim 10$.

PACA_CA has no specific mutated driver gene sets relative to PACA_CA and PAAD when $K = 2 \sim 10$.

Table S1.46: PACA_AU specific mutated driver gene sets relative to PAAD

K	**specific gene set**	p_1	p_2	p
6	*BIRC3,CEBPA,KRAS,CARS,FAM46C,TAF15*	0.026	0.626	0.036
9	*BIRC3,CEBPA,KRAS,CARS,FAM46C,TAF15,MYCN,SOX17,ING1*	0.004	0.854	0.002
10	*BIRC3,CEBPA,KRAS,CARS,FAM46C,TAF15,MYCN,SOX17,ING1,PCBP1*	0.014	0.916	0.005

PACA_AU has no specific mutated driver gene sets relative to PAAD when $K = 2 \sim 10$.

There is no specific driver gene set between PAAD and PACA_CA relative to each other when $K = 2 \sim 10$.

Table S1.47: PACA_AU specific mutated driver gene sets relative to PACA_CA

K	**specific gene set**	p_1	p_2	p
8	*BIRC3,CEBPA,KRAS,CARS,EIF1AX,IFNGR1,RPL10,SOX17*	0.004	1	<0.0001

PACA_CA has no specific mutated driver gene sets relative to PACA_AU when $K = 2 \sim 10$.

Prostate adenocarcinoma

Com3

There is no common driver gene set among PRAD, PRAD_CA and PRAD_UK when $K = 2 \sim 10$.

Com2

There is no common driver gene set between PRAD and PRAD_CA when $K = 2 \sim 10$.

There is no common driver gene set between PRAD and PRAD_UK when $K = 2 \sim 10$.

There is no common driver gene set between PRAD_CA and PRAD_UK when $K = 2 \sim 10$.

Spe

Table S1.48: PRAD specific mutated driver gene sets relative to PRAD_CA and PRAD_UK

K	**specific gene set**	p_1	p_2, p_3	p
3	*TP53,SMARCA1,SPOP*	0.014	0.97,1	<0.0001
4	*TP53,SMARCA1,SPOP,HRAS*	0.013	0.96,1	<0.0001
5	*TP53,SMARCA1,SPOP,HRAS,ZFP36L1*	0.005	0.979,1	<0.0001
6	*TP53,SMARCA1,SPOP,HRAS,ZFP36L1,TAF15*	0.005	0.968,1	<0.0001
7	*TP53,SOX17,SPOP,HRAS,ZFP36L1,TAF15,MAP2K7*	0.009	0.995,1	<0.0001
8	*TP53,SMARCA1,SPOP,HRAS,ZFP36L1,TAF15,MAP2K7,SOX17*	0.002	0.998,1	<0.0001
9	*TP53,SMARCA1,SPOP,HRAS,ZFP36L1,TAF15,MAP2K7,SOX17,MED12*	0.002	1,1	<0.0001
10	*TP53,SMARCA1,SPOP,HRAS,ZFP36L1,TAF15,MAP2K7,SOX17,MED12,SEPT9*	0.001	1,1	<0.0001

Table S1.49: PRAD_UK specific mutated driver gene sets relative to PRAD and PRAD_CA

K	**specific gene set**	p_1	p_2, p_3	p
4	*EHD2,EPHA3,SUZ12,ZNF780A*	<0.0001	0.996,0.998	<0.0001
5	*EHD2,EPHA3,SUZ12,ZNF780A,CNOT9*	<0.0001	0.993,1	<0.0001
6	*EHD2,EPHA3,SUZ12,ZNF780A,CNOT9,ERCC3*	<0.0001	0.994,1	<0.0001
7	*EHD2,EPHA3,SUZ12,ZNF780A,CNOT9,ERCC3,ID3*	<0.0001	0.993,1	<0.0001
8	*EHD2,EPHA3,SUZ12,ZNF780A,CNOT9,ERCC3,ID3,CIC*	<0.0001	0.991,1	<0.0001
9	*BCL9L,DUSP16,EIF1AX,EWSR1,FAM174B,IRAK1,PABPC1,RPL22,STAT5B*	<0.0001	1,1	<0.0001
10	*AXIN2,BIRC3,BTG1,CCR7,CIITA,FOXD4L1,PTCH1,SDC4,SIRPA,TBX3*	<0.0001	1,0.998	<0.0001

PRAD_CA has no specific mutated driver gene sets relative to PRAD and PRAD_UK when $K = 2 \sim 10$.

Table S1.50: PRAD_CA and PRAD_UK specific mutated driver gene sets relative to each other

**Type**	K	**Specific gene set**	p_1	p_2	p
**PRAD_CA / PRAD_UK**	5	*CCR7,LRP1B,NRAS,PTMA,TFAP4*	0.019	1	0.007
	6	*CCR7,LRP1B,NRAS,PTMA,TFAP4,HRAS*	0.03	1	0.006
	7	*CCR7,LRP1B,NRAS,PTMA,TFAP4,HRAS,LTB*	0.016	1	0.003
	8	*CCR7,LRP1B,NRAS,PTMA,TFAP4,HRAS,LTB,LDB1*	0.01	1	<0.0001
	9	*CCR7,LRP1B,NRAS,PTMA,TFAP4,HRAS,LTB,LDB1,NPRL2*	0.01	1	0.001
	10	*CCR7,LRP1B,NRAS,PTMA,TFAP4,HRAS,LTB,LDB1,NPRL2,AMER1*	0.01	1	0.001
**PRAD_UK / PRAD_CA**	2	*EPHA3,TRIM24*	0.006	1	<0.0001
	3	*GATA3,NPM1,PLCB4*	0.011	0.998	<0.0001
	4	*LPAR4,MAX,PDGFRA,RANBP2*	<0.0001	0.924	<0.0001
	5	*TFAP4,HSPG2,SDC4,SIRPA,TBX3*	<0.0001	0.926	<0.0001
	6	*TFAP4,CCR7,GNA11,MAX,SIRPA,TBX3*	<0.0001	0.994	<0.0001
	7	*TFAP4,CCR7,GNA11,MAX,SIRPA,TBX3,ZNF780A*	<0.0001	0.996	<0.0001
	8	*TFAP4,BTG1,CCR7,HSPG2,RSPH10B2,SDC4,SIRPA,TBX3*	<0.0001	0.99	<0.0001
	9	*TFAP4,BTG1,CCR7,HSPG2,RSPH10B2,SDC4,SIRPA,TBX3,CSF3R*	<0.0001	0.999	<0.0001
	10	*TFAP4,BTG1,CCR7,HSPG2,RSPH10B2,SDC4,SIRPA,TBX3,CSF3R,GNA11*	<0.0001	0.999	<0.0001

Skin cutaneous melanoma

Com3

There is no common driver gene set among SKCM, SKCA_BR and SKCM_PCAWG when $K = 2 \sim 10$.

Com2

There is no common driver gene set between SKCM and SKCA_BR when $K = 2 \sim 10$.

Table S1.51: Significant common driver gene set between SKCM and SKCM_PCAWG

K	**common gene set**	p_1	p_2	p
2	*BRAF,NRAS*	<0.0001	<0.0001	<0.0001
3	*BRAF,NRAS,KIT*	<0.0001	<0.0001	<0.0001
4	*BRAF,NRAS,KIT,PTPN11*	<0.0001	<0.0001	<0.0001
5	*BRAF,NRAS,PTPN11,KIT,KRAS*	<0.0001	<0.0001	<0.0001
6	*BRAF,NRAS,PTPN11,KIT,KRAS,HOXA11*	<0.0001	<0.0001	<0.0001
7	*BRAF,NRAS,PTPN11,KIT,KRAS,HOXA11,HRAS*	<0.0001	<0.0001	<0.0001
8	*BRAF,NRAS,PTPN11,KIT,KRAS,HOXA11,HRAS,NT5C3A*	<0.0001	<0.0001	<0.0001
9	*BRAF,NRAS,PTPN11,KIT,KRAS,HOXA11,HRAS,NT5C3A,ZCRB1*	<0.0001	<0.0001	<0.0001
10	*BRAF,NRAS,PTPN11,KIT,KRAS,HOXA11,HRAS,NT5C3A,ZCRB1,CCND1*	<0.0001	<0.0001	<0.0001

There is no common driver gene set between SKCA_BR and SKCM_PCAWG when $K = 2 \sim 10$.

Spe

SKCM has no specific mutated driver gene sets relative to SKCA_BR and SKCM_PCAWG when $K = 2 \sim 10$.

SKCA_BR has no specific mutated driver gene sets relative to SKCM and SKCM_PCAWG when $K = 2 \sim 10$.

SKCM_PCAWG has no specific mutated driver gene sets relative to SKCM and SKCA_BR when $K = 2 \sim 10$.

Table S1.52: SKCM specific mutated driver gene sets relative to SKCA_BR

K	**specific gene set**	p_1	p_2	p
2	*NRAS,BRAF*	<0.0001	0.982	<0.0001
3	*NRAS,BRAF,KIT*	<0.0001	1	<0.0001
4	*NRAS,BRAF,KIT,LY75-CD302*	<0.0001	1	<0.0001
5	*NRAS,BRAF,KIT,LY75-CD302,KRAS*	<0.0001	1	<0.0001
6	*NRAS,BRAF,KIT,LY75-CD302,KRAS,PTPN11*	<0.0001	1	<0.0001
7	*NRAS,BRAF,KIT,LY75-CD302,KRAS,PTPN11,NT5C3A*	<0.0001	1	<0.0001
8	*NRAS,BRAF,KIT,LY75-CD302,KRAS,PTPN11,NT5C3A,RAP1GDS1*	<0.0001	1	<0.0001
9	*NRAS,BRAF,KIT,FAM174B,KRAS,FAM186A,KDM6A,NFE2L2,RBFOX2*	<0.0001	1	<0.0001
10	*NRAS,BRAF,KIT,FAM174B,KRAS,FAM186A,KDM6A,NFE2L2,RBFOX2,NSD2*	<0.0001	1	<0.0001

SKCA_BR has no specific mutated driver gene sets relative to SKCM when $K = 2 \sim 10$.

Table S1.53: SKCM specific mutated driver gene sets relative to SKCM_PCAWG

K	**specific gene set**	p_1	p_2	p
8	*BRD7,CTNNB1,FLCN,LRP1B,PIM1,RNF43,RPL10,UBE2A*	0.037	0.996	0.012
9	*BRD7,CTNNB1,FLCN,LRP1B,PIM1,RNF43,RPL10,UBE2A,SMC1A*	0.022	0.998	0.003
10	*BRD7,CTNNB1,FLCN,LRP1B,PIM1,RNF43,RPL10,UBE2A,SMC1A,DDB2*	0.022	1	0.004

SKCM_PCAWG has no specific mutated driver gene sets relative to SKCM when $K = 2 \sim 10$.

Table S1.54: SKCM_PCAWG specific mutated driver gene sets relative to SKCA_BR

K	**specific gene set**	p_1	p_2	p
2	*BRAF,FBN2*	0.009	1	<0.0001
3	*BRAF,FBN2,ERG*	0.01	1	<0.0001
4	*BRAF,FBN2,LPP,RUNX1*	0.039	1	0.006
5	*BRAF,FBN2,LPP,RUNX1,NRAS*	<0.0001	1	<0.0001
6	*BRAF,FBN2,LPP,RUNX1,NRAS,FHIT*	<0.0001	1	<0.0001
7	*BRAF,FBN2,LPP,RUNX1,NRAS,FHIT,P2RY8*	<0.0001	1	<0.0001
8	*BRAF,MSI2,LPP,RUNX1,NRAS,FHIT,P2RY8,PRKCB*	<0.0001	1	<0.0001
9	*BRAF,MSI2,LPP,RUNX1,NRAS,FHIT,P2RY8,PRKCB,EIF3E*	<0.0001	1	<0.0001
10	*BRAF,MSI2,LPP,RUNX1,NRAS,FHIT,P2RY8,PRKCB,EIF3E,GNAQ*	<0.0001	1	<0.0001

SKCA_BR has no specific mutated driver gene sets relative to SKCM_PCAWG when $K = 2 \sim 10$.

Stomach adenocarcinoma

Com2

There is no common driver gene set between STAD and GACA_CN when $K = 2 \sim 10$.

Spe

Table S1.55: STAD specific mutated driver gene sets relative to GACA_CN

K	**specific gene set**	p_1	p_2	p
2	*TP53,ARID1A*	<0.0001	0.797	<0.0001
3	*TP53,ARID1A,CDH1*	<0.0001	0.992	<0.0001
4	*TP53,ARID1A,CDH1,ESRRA*	<0.0001	0.996	<0.0001
5	*TP53,CSF3R,PTEN,RHOA,SET*	<0.0001	1	<0.0001
6	*TP53,CSF3R,PTEN,RHOA,SET,STAT6*	<0.0001	1	<0.0001
7	*TP53,CSF3R,PTEN,RHOA,SET,STAT6,BCL2L12*	<0.0001	1	<0.0001
8	*TP53,CSF3R,PTEN,RHOA,SET,STAT6,BCL2L12,ZRSR2*	<0.0001	1	<0.0001
9	*TP53,CSF3R,PTEN,RHOA,SET,STAT6,BCL2L12,ZRSR2,CDKN1B*	<0.0001	1	<0.0001
10	*TP53,CSF3R,PTEN,RHOA,SET,STAT6,BCL2L12,ZRSR2,CDKN1B,HRAS*	<0.0001	1	<0.0001

GACA_CN has no specific mutated driver gene sets relative to STAD when $K = 2 \sim 10$.

Thyroid carcinoma

Com2

Table S1.56: Significant common driver gene set between THCA and THCA_SA

K	**common gene set**	p_1	p_2	p
2	*BRAF,NRAS*	<0.0001	<0.0001	<0.0001
3	*BRAF,NRAS,HRAS*	<0.0001	<0.0001	<0.0001
4	*BRAF,NRAS,HRAS,KRAS*	<0.0001	<0.0001	<0.0001
5	*TP53,BRAF,NRAS,HRAS,KRAS*	<0.0001	<0.0001	<0.0001
6	*TP53,BRAF,NRAS,KRAS,HRAS,EIF1AX*	<0.0001	<0.0001	<0.0001
7	*TP53,BRAF,NRAS,HRAS,KRAS,EIF1AX,HLA-B*	<0.0001	<0.0001	<0.0001
8	*TP53,BRAF,NRAS,HRAS,KRAS,EIF1AX,LATS2,SIRPA*	<0.0001	<0.0001	<0.0001
9	*TP53,BRAF,NRAS,HRAS,KRAS,EIF1AX,LATS2,SIRPA,HLA-B*	<0.0001	<0.0001	<0.0001
10	*TP53,BRAF,NRAS,HRAS,KRAS,EIF1AX,LATS2,SIRPA,HLA-B,WT1*	<0.0001	<0.0001	<0.0001

Spe

There is no specific driver gene set between THCA and THCA_SA relative to each other when $K = 2 \sim 10$.

2 Tables: Pathway analysis of the tissues with similar location or related functions

Breast adenocarcinoma vs Ovarian cancer

There is no common driver gene set between BRCA and OV when $K = 2 \sim 10$.

Table S2.1: BRCA and OV specific mutated driver gene sets relative to each other

Type	K	Specific gene set	p_1	p_2	p
BRCA / OV	2	*GATA3,PIK3CA*	0.002	0.996	0.004
	3	*GATA3,PIK3CA,PTEN*	0.002	0.853	<0.0001
	4	*GATA3,PIK3CA,PTEN,MED12*	0.999	1	<0.0001
	5	*GATA3,PIK3CA,PTEN,MED12,RABEP1*	<0.0001	1	<0.0001
	6	*GATA3,PIK3CA,PTEN,MED12,RABEP1,CARD11*	<0.0001	1	<0.0001
	7	*GATA3,PIK3CA,PTEN,MED12,RABEP1,CARD11,RUNX1*	<0.0001	1	<0.0001
	8	*GATA3,PIK3CA,PTEN,MED12,RABEP1,CARD11,RUNX1,IL6ST*	<0.0001	0.999	<0.0001
	9	*GATA3,PIK3CA,PTEN,MED12,RABEP1,CARD11,RUNX1,IL6ST,PIK3R1*	<0.0001	0.994	<0.0001
	10	*GATA3,PIK3CA,PTEN,MED12,RABEP1,CARD11,RUNX1,IL6ST,PIK3R1,PLCG1*	<0.0001	0.993	<0.0001
OV / BRCA	3	*TP53,CBL,RBM10*	0.008	1	<0.0001
	4	*TP53,CBL,RBM10,NFKBIE*	0.001	1	<0.0001
	5	*TP53,CBL,FAM46C,NFKBIE,U2AF2*	<0.0001	1	<0.0001
	6	*TP53,CBL,FAM46C,NFKBIE,U2AF2,BCL10*	<0.0001	1	<0.0001
	7	*TP53,CBL,FAM46C,NFKBIE,U2AF2,BCL10,MAF*	<0.0001	1	<0.0001
	8	*TP53,CBL,FAM46C,NFKBIE,U2AF2,BCL10,MAF,CCND1*	<0.0001	1	<0.0001
	9	*TP53,CBL,FAM46C,NFKBIE,U2AF2,BCL10,MAF,CCND1,CD58*	<0.0001	1	<0.0001
	10	*TP53,CBL,FAM46C,NFKBIE,U2AF2,BCL10,MAF,CCND1,CD58,RGL3*	<0.0001	1	<0.0001

Table S2.2: Significant common driver gene set between BRCA_UK and OV

K	**common gene set**	p_1	p_2	p
6	*TP53,EIF1AX,FAM46C,GATA3,PIK3CA,RAC1*	0.028	0.025	0.007
7	*TP53,EIF1AX,FAM46C,GATA3,PIK3CA,RAC1,ACKR3*	0.021	0.003	<0.0001
8	*TP53,EIF1AX,FAM46C,GATA3,PIK3CA,RAC1,ACKR3,FGFR3*	0.01	0.01	0.001
9	*TP53,EIF1AX,FAM46C,GATA3,PIK3CA,RAC1,ACKR3,FGFR3,LOX*	0.01	0.006	0.001
10	*TP53,EIF1AX,FAM46C,GATA3,PIK3CA,RAC1,ACKR3,FGFR3,LOX,CD79B*	0.013	0.006	<0.0001

Table S2.3: OV specific mutated driver gene sets relative to BRCA_UK

K	**specific gene set**	p_1	p_2	p
6	*TP53,EIF1AX,FAM46C,GATA3,PIK3CA,RAC1*	0.028	0.025	0.007
7	*TP53,EIF1AX,FAM46C,GATA3,PIK3CA,RAC1,ACKR3*	0.021	0.003	<0.0001
8	*TP53,EIF1AX,FAM46C,GATA3,PIK3CA,RAC1,ACKR3,FGFR3*	0.01	0.01	0.001
9	*TP53,EIF1AX,FAM46C,GATA3,PIK3CA,RAC1,ACKR3,FGFR3,LOX*	0.01	0.006	0.001
10	*TP53,EIF1AX,FAM46C,GATA3,PIK3CA,RAC1,ACKR3,FGFR3,LOX,CD79B*	0.013	0.006	<0.0001

BRCA_UK has no specific mutated driver gene sets relative to OV when $K = 2 \sim 10$.

Lung adenocarcinoma vs Lung squamous cell carcinoma

Table S2.4: Significant common driver gene set between LUAD and LUSC

K	**common gene set**	p_1	p_2	p
2	*TP53,KRAS*	0.001	0.036	<0.0001
3	*TP53,KRAS,MET*	<0.0001	0.034	<0.0001
4	*TP53,KRAS,MET,CTNNB1*	<0.0001	0.037	<0.0001
5	*TP53,KRAS,MET,CTNNB1,PTPN6*	<0.0001	0.016	<0.0001
6	*TP53,KRAS,MET,CTNNB1,PTPN6,NPRL2*	<0.0001	0.027	<0.0001
7	*TP53,KRAS,MET,CTNNB1,PTPN6,NPRL2,MYD88*	<0.0001	0.008	<0.0001
8	*TP53,KRAS,MET,CTNNB1,PTPN6,NPRL2,MYD88,IRF1*	<0.0001	0.003	<0.0001
9	*TP53,KRAS,MET,CTNNB1,PTPN6,NPRL2,MYD88,IRF1,USP44*	<0.0001	0.003	<0.0001
10	*TP53,KRAS,MET,CTNNB1,PTPN6,NPRL2,MYD88,IRF1,USP44,PRKCD*	<0.0001	<0.0001	<0.0001

Table S2.5: LUAD and LUSC specific mutated driver gene sets relative to each other

Type	K	Specific gene set	p_1	p_2	p
**LUAD / LUSC**	2	*EGFR,LRP1B*	0.007	0.999	<0.0001
	3	*EGFR,STK11,PTPRD*	0.001	0.699	0.001
	4	*EGFR,STK11,PTPRD,KDM5C*	<0.0001	0.447	<0.0001
	5	*EGFR,STK11,PTPRD,KDM5C,SMARCA4*	<0.0001	0.898	<0.0001
	6	*EGFR,STK11,PTPRD,KDM5C,PPT2,SMAD2*	<0.0001	0.734	<0.0001
	7	*EGFR,STK11,CMTR2,PDGFRB,PML,SMARCA4,SOHLH2*	<0.0001	0.998	<0.0001
	8	*EGFR,STK11,CMTR2,PDGFRB,PML,SMARCA4,SOHLH2,ELF3*	<0.0001	1	<0.0001
	9	*EGFR,STK11,CMTR2,PDGFRB,PML,SMARCA4,SOHLH2,ELF3,SMC1A*	<0.0001	1	<0.0001
	10	*EGFR,STK11,CMTR2,PDGFRB,PML,SMARCA4,SOHLH2,ELF3,SMC1A,FOXD4L1*	<0.0001	1	<0.0001
**LUSC / LUAD**	3	*KMT2D,NFE2L2,PTEN*	0.045	0.69	0.045
	4	*KMT2D,NFE2L2,PTEN,SGK1*	0.003	0.948	0.008
	5	*KMT2D,NFE2L2,PTEN,SGK1,RASA1*	0.004	0.897	0.001
	6	*KMT2D,NFE2L2,PTEN,SGK1,RASA1,H3F3A*	<0.0001	0.91	0.001
	7	*FOXL2,H3F3A,KMT2D,NFE2L2,PTEN,RASA1,SGK1*	<0.0001	0.958	0.001
	8	*FOXL2,H3F3A,KMT2D,NFE2L2,PTEN,RASA1,SGK1,SUZ12*	0.001	0.963	0.001
	9	*FOXL2,FUBP1,H3F3A,KMT2D,NFE2L2,PTEN,RASA1,SGK1,SUZ12*	0.001	0.985	<0.0001
	10	*CDX2,FOXL2,FUBP1,H3F3A,KMT2D,NFE2L2,PTEN,RASA1,SGK1,SUZ12*	<0.0001	0.992	<0.0001

Esophageal cancer vs Stomach adenocarcinoma

Table S2.6: Significant common driver gene set between ESCA and STAD

K	**common gene set**	p_1	p_2	p
4	*TP53,ARID1A,CDH1,LDB1*	0.014	<0.0001	<0.0001
5	*TP53,ARID1A,CDH1,LDB1,ACVR1*	0.009	<0.0001	<0.0001
6	*TP53,ARID1A,CDH1,LDB1,ACVR1,UBE2D2*	0.001	<0.0001	<0.0001
7	*TP53,ARID1A,CDH1,LDB1,ACVR1,UBE2D2,PCBP1*	0.001	<0.0001	<0.0001
8	*TP53,ARID1A,CDH1,LDB1,ACVR1,UBE2D2,PCBP1,CDK4*	<0.0001	<0.0001	<0.0001
9	*TP53,ARID1A,CDH1,LDB1,ACVR1,UBE2D2,PCBP1,CDK4,CXCR4*	0.004	<0.0001	<0.0001
10	*TP53,ARID1A,CDH1,LDB1,ACVR1,UBE2D2,PCBP1,CDK4,CXCR4,BCL2L12*	<0.0001	<0.0001	<0.0001

Table S2.7: ESCA and STAD specific mutated driver gene sets relative to each other

**Type**	K	**Specific gene set**	p_1	p_2	p
**ESCA / STAD**	2	*TP53,SOX9*	0.001	0.825	0.001
	3	*TP53,PRKAR1A,SETD1B*	0.001	0.981	<0.0001
	7	*FAM186A,MAF,NFE2L2,NPEPPS,PCMTD1,RGS7,CXCR4*	0.035	1	<0.0001
	8	*FAM186A,MAF,NFE2L2,NPEPPS,PCMTD1,RGS7,CXCR4,AJUBA*	0.025	1	<0.0001
	9	*FAM186A,MAF,NFE2L2,NPEPPS,PCMTD1,RGS7,CEBPA,AJUBA,USP6*	0.022	1	<0.0001
	10	*FAM186A,MAF,NFE2L2,NPEPPS,PCMTD1,RGS7,CXCR4,AJUBA,USP6,CEBPA*	0.009	1	<0.0001
**STAD / ESCA**	2	*TP53,ARID1A*	<0.0001	0.074	<0.0001
	3	*TP53,ARID1A,NFE2L2*	<0.0001	1	<0.0001

Colorectal adenocarcinoma vs Liver hepatocellular carcinoma

Table S2.8: Significant common driver gene set between COADREAD and LIHC

K	**common gene set**	p_1	p_2	p
4	*TP53,BAP1,CTNNB1,EHD2*	0.006	<0.0001	<0.0001
5	*TP53,BAP1,CTNNB1,EHD2,AXIN1*	0.009	<0.0001	<0.0001
6	*TP53,BAP1,CTNNB1,EHD2,AXIN1,IRAK1*	0.002	<0.0001	<0.0001
7	*TP53,BAP1,CTNNB1,EHD2,AXIN1,IRAK1,CCND2*	0.004	<0.0001	<0.0001
8	*TP53,BAP1,CTNNB1,EHD2,AXIN1,IRAK1,CCND2,CCND3*	0.002	<0.0001	<0.0001
9	*TP53,BAP1,CTNNB1,DDX6,AXIN1,IRAK1,CCND2,CCND3,PPP2R1A*	<0.0001	<0.0001	<0.0001
10	*TP53,BAP1,CTNNB1,RAC1,AXIN1,IRAK1,CCND2,CCND3,PPP2R1A,MTCP1*	<0.0001	<0.0001	<0.0001

Table S2.9: COADREAD and LIHC specific mutated driver gene sets relative to each other

**Type**	K	**Specific gene set**	p_1	p_2	p
**COADREAD / LIHC**	2	APC,BRAF	<0.0001	1	<0.0001
	3	APC,BRAF,CNOT9	<0.0001	1	<0.0001
	4	APC,BRAF,BCL2L12,TFAP4	<0.0001	1	<0.0001
	5	APC,BRAF,BCL2L12,TFAP4,FHIT	<0.0001	1	<0.0001
	6	APC,BMPR1A,DGCR8,CCND3,DAXX,ATG7	<0.0001	0.848	<0.0001
	7	APC,BMPR1A,DGCR8,CCND3,DAXX,ATG7,BCL2L12	<0.0001	0.849	<0.0001
	8	APC,BMPR1A,DGCR8,CD79B,CRTC1,FHIT,BCL2L12,PSIP1	<0.0001	1	<0.0001
	9	APC,BMPR1A,DGCR8,CD79B,CRTC1,FHIT,BCL2L12,PSIP1,TFAP4	<0.0001	1	<0.0001
	10	APC,BMPR1A,DGCR8,CD79B,CRTC1,FHIT,BCL2L12,PSIP1,TFAP4,CNOT9	<0.0001	1	<0.0001
**LIHC / COADREAD**	2	AXIN1,CTNNB1	0.005	0.987	0.001
	3	AXIN1,CTNNB1,BAP1	<0.0001	0.999	<0.0001

Kidney renal clear cell carcinoma vs Prostate adenocarcinoma

There is no common driver gene set between KIRC and PRAD when $K = 2 \sim 10$.

Table S2.10: KIRC and PRAD specific mutated driver gene sets relative to each other

**Type**	K	**Specific gene set**	p_1	p_2	p
**KIRC / PRAD**	4	*BCORL1,RARA,STAG2,VHL*	0.014	1	0.004
	5	*BCORL1,RARA,STAG2,VHL,BRCA2*	<0.0001	1	<0.0001
	6	*BCORL1,RARA,STAG2,VHL,UBR5,HSP90AA1*	0.001	1	0.001
	7	*BCORL1,RARA,STAG2,VHL,UBR5,HSP90AA1,NKTR*	<0.0001	1	<0.0001
	8	*BCORL1,RARA,STAG2,VHL,UBR5,HSP90AA1,NKTR,ERG*	<0.0001	1	<0.0001
	9	*BCORL1,RARA,STAG2,VHL,UBR5,HSP90AA1,NKTR,ERG,FLCN*	<0.0001	1	<0.0001
	10	*BCORL1,RARA,STAG2,VHL,UBR5,HSP90AA1,NKTR,ERG,FLCN,BRCA2*	<0.0001	1	<0.0001
**PRAD / KIRC**	7	*TP53,CDKN1B,FOXA1,KDM6A,PTPRC,SMARCA1,SPOP*	0.025	0.937	0.016
	8	*TP53,CDKN1B,FOXA1,KDM6A,PTPRC,SMARCA1,SPOP,HRAS*	0.009	0.925	0.017
	9	*TP53,CDKN1B,FOXA1,KDM6A,PTPRC,SMARCA1,SPOP,HRAS,PREX2*	0.007	0.899	0.009
	10	*TP53,CDKN1B,FOXA1,KDM6A,PTPRC,SMARCA1,SPOP,HRAS,PCDH17,CAMTA1*	0.001	0.892	0.006

Kidney renal clear cell carcinoma vs Bladder cancer

Table S2.11: Significant common driver gene set between KIRC and BLCA

K	**common gene set**	p_1	p_2	p
2	*TP53,ARID1A*	0.002	<0.0001	<0.0001
3	*TP53,ARID1A,SOCS1*	<0.0001	0.002	<0.0001
4	*TP53,EBF1,RHOA,CDH1*	0.014	<0.0001	<0.0001
5	*TP53,EBF1,RHOA,CDH1,ATG7*	0.009	<0.0001	<0.0001
6	*TP53,EBF1,RHOA,ATG7,CDKN1B,HOXD13*	0.001	<0.0001	<0.0001
7	*TP53,EBF1,RHOA,IDH1,CDKN1B,HOXD13,STAT6*	0.001	<0.0001	<0.0001
8	*TP53,EBF1,RHOA,ATG7,CDKN1B,HOXD13,STAT6,CD79B*	<0.0001	<0.0001	<0.0001
9	*TP53,EBF1,RHOA,ATG7,CDKN1B,HOXD13,STAT6,CD79B,BCL2L12*	0.004	<0.0001	<0.0001
10	*TP53,EBF1,RHOA,ATG7,CDKN1B,HOXD13,STAT6,CD79B,BCL2L12,CXCR4*	<0.0001	<0.0001	<0.0001

Table S2.12: KIRC and BLCA specific mutated driver gene sets relative to each other

**Type**	K	**Specific gene set**	p_1	p_2	p
**KIRC / BLCA**	5	*BCL2L12,GNA11,MAX,SETD1B,VHL*	0.038	0.999	0.006
	6	*BCL2L12,GNA11,MAX,SETD1B,VHL,PLAG1*	0.019	1	0.002
	7	*BAP1,CUL3,FGFR1,KDM5C,MSN,PBRM1,SETD1B*	0.003	1	<0.0001
	8	*BAP1,CUL3,FGFR1,KDM5C,MSN,PBRM1,SETD1B,XPC*	<0.0001	1	<0.0001
	9	*BAP1,CUL3,FGFR1,KDM5C,MSN,PBRM1,SETD1B,XPC,NPM1*	0.002	1	<0.0001
	10	*BAP1,CUL3,FGFR1,KDM5C,MSN,PBRM1,SETD1B,XPC,P2RY8,RNF43*	<0.0001	1	<0.0001
**BLCA / KIRC**	2	*TP53,FGFR3*	<0.0001	0.951	<0.0001
	3	*TP53,FGFR3,HRAS*	<0.0001	0.961	<0.0001
	4	*TP53,FGFR3,HRAS,FGFR2*	<0.0001	0.965	<0.0001
	5	*TP53,FGFR3,HRAS,FGFR2,KRAS*	<0.0001	0.916	<0.0001
	6	*TP53,FGFR3,HRAS,FGFR2,KRAS,HLA-B*	<0.0001	0.917	<0.0001
	7	*DDB2,HOXC13,ING1,KDM6A,KMT2D,RB1,SATB1*	<0.0001	0.931	<0.0001
	8	*DDB2,HOXC13,ING1,KDM6A,KMT2D,RB1,SATB1,EXT2*	<0.0001	0.922	<0.0001
	9	*DDB2,HOXC13,ING1,KDM6A,KMT2D,RB1,SATB1,EXT2,GATA1*	<0.0001	0.944	<0.0001
	10	*DDB2,HOXC13,ING1,KDM6A,KMT2D,RB1,SATB1,EXT2,GATA1,U2AF1*	<0.0001	0.943	<0.0001

Liver hepatocellular carcinoma vs Pancreatic adenocarcinoma

There is no common driver gene set between LIHC and PAAD when $K = 2 \sim 10$.

Table S2.13: ESCA and ESCA_CN specific mutated driver gene sets relative to each other

Type	K	Specific gene set	p_1	p_2	p
LIHC / PAAD	2	*TP53,CTNNB1*	0.019	0.637	0.024
	3	*AXIN1,BAP1,CTNNB1*	<0.0001	1	<0.0001
	4	*AXIN1,BAP1,CTNNB1,RB1*	<0.0001		<0.0001
	5	*AXIN1,BAP1,CTNNB1,RB1,HNF1A*	<0.0001	1	<0.0001
	6	*AXIN1,BAP1,CTNNB1,RB1,HNF1A,NFATC2*	<0.0001	1	0.001
	7	*AXIN1,BAP1,CTNNB1,RB1,HNF1A,NFATC2,BIRC6*	<0.0001	1	<0.0001
	8	*AXIN1,BAP1,CTNNB1,RB1,HNF1A,NCOR1,BIRC6,NFATC2*	<0.0001	1	<0.0001
	9	*AXIN1,BAP1,CTNNB1,RB1,HNF1A,NCOR1,BIRC6,NFATC2,RECQL4*	<0.0001	1	<0.0001
	10	*AXIN1,BAP1,CTNNB1,RB1,HNF1A,NCOR1,BIRC6,NFATC2,RECQL4,USP8*	<0.0001	1	<0.0001
PAAD / LIHC	4	*BRAF,KRAS,PRKCD,SOX17*	0.035	1	0.046
	5	*BRAF,KRAS,PRKCD,SOX17,FHIT*	0.016	1	0.017
	6	*BRAF,KRAS,PRKCD,SOX17,FHIT,FOXA2*	0.006	1	0.01
	7	*BRAF,KRAS,PRKCD,SOX17,FHIT,FOXA2,CD79B*	0.004	0.985	0.003
	8	*BRAF,KRAS,PRKCD,SOX17,FHIT,FOXA2,CD79B,CRNKL1*	0.001	0.972	0.003
	9	*BRAF,KRAS,PRKCD,SOX17,FHIT,FOXA2,CD79B,CRNKL1,ABI1*	0.002	0.958	0.003
	10	*BRAF,KRAS,PRKCD,SOX17,FHIT,FOXA2,CD79B,CRNKL1,ABI1,NPRL2*	0.004	0.965	<0.0001

Colorectal adenocarcinoma vs Stomach adenocarcinoma

Table S2.14: Significant common driver gene set between COADREAD and STAD

K	**common gene set**	p_1	p_2	p
2	*TP53,ARID1A*	0.002	<0.0001	<0.0001
3	*TP53,ARID1A,SOCS1*	<0.0001	0.002	<0.0001
4	*TP53,CDH1,EBF1,RHOA*	<0.0001	<0.0001	<0.0001
5	*TP53,CDH1,EBF1,RHOA,ATG7*	<0.0001	<0.0001	<0.0001
6	*TP53,ATG7,EBF1,RHOA,CDKN1B,HOXD13*	<0.0001	<0.0001	<0.0001
7	*TP53,IDH1,EBF1,RHOA,CDKN1B,HOXD13,STAT6*	<0.0001	<0.0001	<0.0001
8	*TP53,ATG7,EBF1,RHOA,CDKN1B,HOXD13,STAT6,CD79B*	<0.0001	<0.0001	<0.0001
9	*TP53,ATG7,EBF1,RHOA,CDKN1B,HOXD13,STAT6,CD79B,BCL2L12*	<0.0001	<0.0001	<0.0001
10	*TP53,ATG7,EBF1,RHOA,CDKN1B,HOXD13,STAT6,CD79B,BCL2L12,CXCR4*	<0.0001	<0.0001	<0.0001

Table S2.15: COADREAD specific mutated driver gene sets relative to STAD

K	**specific gene set**	p_1	p_2	p
2	*APC,BRAF*	<0.0001	0.866	<0.0001
3	*APC,CD79B,CRTC1*	<0.0001	0.997	<0.0001
4	*APC,CD79B,CRTC1,SOCS1*	<0.0001	0.997	<0.0001
5	*APC,CD79B,CRTC1,SOCS1,CDX2*	<0.0001	0.997	<0.0001
6	*APC,CD79B,CRTC1,SOCS1,CDX2,CNOT9*	<0.0001	1	<0.0001
7	*APC,CCND3,CDKN1A,SOCS1,FOXA1,CNOT9,AJUBA*	<0.0001	1	<0.0001
8	*APC,CCND3,CDKN1A,SOCS1,FOXA1,CNOT9,AJUBA,UBE2D2*	<0.0001	1	<0.0001
9	*APC,CCND3,CDKN1A,SOCS1,FOXA1,CNOT9,AJUBA,UBE2D2,PTPN6*	<0.0001	1	<0.0001
10	*APC,CCND3,CDKN1A,SOCS1,FOXA1,CNOT9,AJUBA,UBE2D2,CDX2,EIF1AX*	<0.0001	1	<0.0001

STAD has no specific mutated driver gene sets relative to COADREAD when $K = 2 \sim 10$.

Thyroid carcinoma vs Breast, Pancreatic and Prostate adenocarcinoma

There is no common driver gene set between THCA and BRCA, PAAD, PRAD when $K = 2 \sim 10$.

Table S2.16: THCA specific mutated driver gene sets relative to BRCA, PAAD, PRAD

K	**specific gene set**	p_1	p_2, p_3, p_4	p
2	*BRAF,NRAS*	<0.0001	1,0.99,1	<0.0001
3	*BRAF,NRAS,HRAS*	<0.0001	0.964,0.999,0.996	<0.0001
4	*BRAF,NRAS,HRAS,EIF1AX*	<0.0001	0.964,0.998,0.994	<0.0001
5	*BRAF,NRAS,HRAS,EIF1AX,PPP6C*	<0.0001	0.975,0.999,1	<0.0001
6	*BRAF,NRAS,HRAS,EIF1AX,PPP6C,BCL2L12*	<0.0001	0.968,0.992,1	<0.0001
7	*BRAF,NRAS,HRAS,EIF1AX,PPP6C,BCL2L12,MAP2K1*	<0.0001	0.964,1,1	<0.0001
8	*BRAF,NRAS,HRAS,EIF1AX,PPP6C,BCL2L12,MAP2K1,ALB*	<0.0001	0.969,1,1	<0.0001
9	*BRAF,NRAS,HRAS,EIF1AX,PPP6C,BCL2L12,MAP2K1,ALB,FBXO11*	<0.0001	0.964,1,1	<0.0001
10	*BRAF,NRAS,HRAS,EIF1AX,PPP6C,BCL2L12,MAP2K1,ALB,FBXO11,WDR45*	<0.0001	0.968,1,1	<0.0001

Glioblastoma multiforme vs Head and neck squamous cell carcinoma

There is no common driver gene set between GBM and HNSC when $K = 2 \sim 10$.

Table S2.17: GBM and HNSC specific mutated driver gene sets relative to each other

**Type**	K	**Specific gene set**	p_1	p_2	p
**GBM / HNSC**	2	*IDH1,PTEN*	<0.0001	0.99	<0.0001
	4	*IDH1,PTEN,EGFR,KEL*	0.029	0.846	0.028
	5	*IDH1,PTEN,EGFR,KEL,NF1*	0.007	0.999	0.003
	6	*IDH1,PTEN,EGFR,KEL,NF1,DDX6*	0.006	1	0.001
	7	*IDH1,PTEN,EGFR,KEL,NF1,DDX6,WAS*	0.004	1	<0.0001
	8	*IDH1,PTEN,EGFR,KEL,NF1,DDX6,WAS,CCND2*	0.003	1	<0.0001
	9	*IDH1,PTEN,EGFR,KEL,NF1,DDX6,WAS,CCND2,H3F3A*	0.004	1	<0.0001
	10	*IDH1,PTEN,EGFR,KEL,NF1,DDX6,WAS,CCND2,H3F3A,NFKBIE*	0.002	1	<0.0001
**HNSC / GBM**	2	*TP53,RB1*	0.013	1	<0.0001
	3	*TP53,HRAS,IDH1*	<0.0001	1	<0.0001
	4	*TP53,HRAS,IDH1,FGFR3*	<0.0001	1	<0.0001
	5	*TP53,HRAS,IDH1,FGFR3,PMS2*	<0.0001	1	<0.0001
	6	*TP53,CDKN1B,CYLD,FGFR3,HRAS,PMS2*	<0.0001	1	<0.0001
	7	*TP53,CDKN1B,CYLD,FGFR3,HRAS,PMS2,RASA2*	<0.0001	1	<0.0001
	8	*TP53,CDKN1B,CYLD,FGFR3,HRAS,PMS2,RASA2,SMO*	<0.0001	1	<0.0001
	9	*TP53,CDKN1B,CYLD,FGFR3,HRAS,PMS2,RASA2,SMO,RELA*	<0.0001	1	<0.0001
	10	*TP53,CDKN1B,CYLD,FGFR3,HRAS,PMS2,RASA2,SMO,IRAK1,RBM10*	<0.0001	1	<0.0001

3 Tables: The signaling profile for kids

Glioblastoma multiforme

Table S3.1: Significant common driver gene set between GBM and GBM_DE

K	**common gene set**	p_1	p_2	p
9	*CASP8,H3F3A,IDH1,LPAR4,LRP1B,MAP2K7,PIK3CA,PTEN,KLHL6*	<0.0001	0.018	<0.0001
10	*CASP8,H3F3A,IDH1,LPAR4,LRP1B,MAP2K7,PIK3CA,PTEN,PTMA,ZFP36L1*	<0.0001	0.042	<0.0001

Table S3.2: Significant common driver gene set between GBM_MSKCC and GBM_DE

K	**common gene set**	p_1	p_2	p
7	*TP53,DCC,EGFR,NF1,RBM38,RPS3A,WAS*	<0.0001	0.02	<0.0001
8	*TP53,DCC,EGFR,NF1,RBM38,RPS3A,WAS,KLHL6*	<0.0001	0.006	<0.0001
9	*TP53,DCC,EGFR,NF1,RBM38,RPS3A,WAS,KLHL6,EIF1AX*	<0.0001	0.009	<0.0001
10	*TP53,DCC,EGFR,NF1,RBM38,RPS3A,WAS,KLHL6,EIF1AX,SOCS1*	<0.0001	0.003	<0.0001

There is no common driver gene set among GBM, GBM_MSKCC and GBM_DE when $K = 2 \sim 10$.

Table S3.3: GBM_DE specific mutated driver gene sets relative to GBM and GBM_MSKCC

K	**specific gene set**	p_1	p_2, p_3	p
2	*PTPRD,RABEP1*	0.024	1,1	<0.0001
3	*PTPRD,RABEP1,RBM38*	0.003	1,1	0.015
4	*PTPRD,RABEP1,RBM38,CEBPA*	0.002	1,1	<0.0001
5	*PTPRD,RABEP1,RBM38,CEBPA,PRF1*	<0.0001	1,1	<0.0001
6	*PTPRD,RABEP1,RBM38,CEBPA,PRF1,WDR45*	<0.0001	1,1	<0.0001
8	*PTPRD,RABEP1,RBM38,CEBPA,PRF1,WDR45,ID3,SMO*	<0.0001	1,1	<0.0001
9	*PTPRD,RABEP1,RBM38,CEBPA,PRF1,WDR45,ID3,SMO,PTMA*	<0.0001	1,1	<0.0001

Table S3.4: GBM and GBM_DE specific mutated driver gene sets relative to each other

**Type**	K	**Specific gene set**	p_1	p_2	p
**GBM / GBM_DE**	3	*IDH1,PIK3CA,PTEN*	<0.0001	0.706	0.001
	4	*IDH1,PIK3CA,PTEN,H3F3A*	0.001	0.695	<0.0001
	5	*IDH1,PIK3CA,PTEN,H3F3A,LPAR4*	<0.0001	0.685	<0.0001
	6	*IDH1,PIK3CA,PTEN,H3F3A,LPAR4,KMT2B*	<0.0001	0.968	<0.0001
	7	*IDH1,PIK3CA,PTEN,H3F3A,LPAR4,KMT2B,WAS*	<0.0001	0.986	<0.0001
	8	*IDH1,PIK3CA,PTEN,H3F3A,LPAR4,KMT2B,WAS,EIF1AX*	<0.0001	0.978	<0.0001
	9	*TP53,CDKN2A,DIS3,EGFR,FGFR4,IRAK1,KDM3B,KEL,WAS*	0.001	0.992	0.001
	10	*TP53,CDKN2A,DIS3,EGFR,FGFR4,IRAK1,KDM3B,KEL,WAS,SOX21*	0.001	0.992	<0.0001
**GBM_DE / GBM**	2	*PTPRD,RABEP1*	0.019	1	0.002
	3	*PTPRD,RABEP1,SMO*	0.007	1	<0.0001
	4	*PTPRD,PPP2R1A,RBM38,ZNF721*	<0.0001	1	<0.0001
	5	*PTPRD,PPP2R1A,RBM38,ZNF721,GNA11*	<0.0001	1	<0.0001
	6	*PTPRD,PPP2R1A,RBM38,ZNF721,GNA11,KLHL36*	<0.0001	1	<0.0001
	7	*PTPRD,PPP2R1A,RBM38,ZNF721,GNA11,ID3,PRF1*	<0.0001	1	<0.0001
	8	*PTPRD,PPP2R1A,RBM38,ZNF721,GNA11,ID3,PRF1,KLHL36*	<0.0001	1	<0.0001
	9	*PTPRD,PPP2R1A,RBM38,CDK4,CEBPA,GNA11,ID3,PRF1,SMO*	<0.0001	1	<0.0001
	10	*PTPRD,PPP2R1A,RBM38,CDK4,CEBPA,GNA11,ID3,PRF1,SMO,WDR45*	<0.0001	1	<0.0001

Table S3.5: GBM_MSKCC and GBM_DE specific mutated driver gene sets relative to each other

Type	K	Specific gene set	p_1	p_2	p
**GBM_MSKCC / GBM_DE**	2	*TP53,EGFR*	<0.0001	0.713	<0.0001
	3	*TP53,EGFR,NF1*	<0.0001	0.843	<0.0001
	4	*H3F3A,IDH1,PIK3R1,PTEN*	<0.0001	0.88	<0.0001
	5	*H3F3A,IDH1,PIK3R1,PTEN,JAK3*	<0.0001	0.97	<0.0001
	6	*H3F3A,IDH1,PIK3R1,PTEN,JAK3,EIF1AX*	<0.0001	0.973	<0.0001
	7	*H3F3A,IDH1,PIK3R1,PTEN,JAK3,EIF1AX,CLTC*	<0.0001	0.999	<0.0001
	8	*H3F3A,IDH1,PIK3R1,PTEN,JAK3,EIF1AX,CLTC,HNRNPA2B1*	<0.0001	1	<0.0001
	9	*H3F3A,IDH1,PIK3R1,PTEN,JAK3,CCR7,CLTC,HNRNPA2B1,KMT2B*	<0.0001	1	<0.0001
	10	*H3F3A,IDH1,PIK3R1,PTEN,JAK3,BCL2L12,CLTC,HNRNPA2B1,KMT2B,ZFP36L1*	<0.0001	1	<0.0001
**GBM_DE / GBM_MSKCC**	2	*PTPRD,RABEP1*	0.026	1	0.016
	3	*PTPRD,RABEP1,RBM38*	0.002	1	0.002
	4	*PTPRD,RABEP1,RBM38,CEBPA*	<0.0001	1	<0.0001
	5	*PTPRD,RABEP1,RBM38,CEBPA,RGL3*	<0.0001	1	<0.0001
	6	*PTPRD,RABEP1,RBM38,CEBPA,CHD4,MAP2K7*	<0.0001	1	<0.0001
	7	*PTPRD,RABEP1,PTMA,CEBPA,CHD4,MAP2K7,UGT2B17*	<0.0001	1	<0.0001
	8	*PTPRD,RABEP1,PTMA,CEBPA,CHD4,UGT2B17,DCAF12L2,RGL3*	<0.0001	1	<0.0001
	9	*PTPRD,RABEP1,PTMA,CEBPA,PRF1,UGT2B17,DCAF12L2,RGL3,WDR45*	<0.0001	1	<0.0001
	10	*PTPRD,RABEP1,PTMA,CEBPA,PRF1,UGT2B17,DCAF12L2,RGL3,WDR45,HIST1H3B*	<0.0001	1	<0.0001

Acute myeloid leukemia

Table S3.6: Significant common driver gene set between LAML and AML_TARGET

K	**common gene set**	p_1	p_2	p
5	*TP53,FLT3,KIT,KRAS,NRAS*	0.021	<0.0001	<0.0001
6	*FLT3,KIT,KRAS,NRAS,PTPN11,RUNX1*	0.001	<0.0001	<0.0001
7	*TP53,FLT3,KIT,KRAS,NRAS,PTPN11,RUNX1*	<0.0001	<0.0001	<0.0001
8	*TP53,FLT3,KIT,KRAS,NRAS,PTPN11,RUNX1,WT1*	0.003	<0.0001	<0.0001
9	*TP53,FLT3,KIT,KRAS,NRAS,PTPN11,RUNX1,WT1,GATA2*	<0.0001	<0.0001	<0.0001
10	*TP53,FLT3,KIT,KRAS,NRAS,PTPN11,RUNX1,WT1,GATA2,TET2*	<0.0001	<0.0001	<0.0001

There is no common driver gene set between LAML_CN and AML_TARGET when $K = 2 \sim 10$.

There is no common driver gene set between LAML_KR and AML_TARGET when $K = 2 \sim 10$.

There is no common driver gene set among LAML, LAML_CN, LAML_KR and AML_TARGET when $K = 2 \sim 10$.

There is no common driver gene set among LAML_CN, LAML_KR and AML_TARGET when $K = 2 \sim 10$.

There is no common driver gene set among LAML, LAML_KR and AML_TARGET when $K = 2 \sim 10$.

There is no common driver gene set among LAML, LAML_CN and AML_TARGET when $K = 2 \sim 10$.

Table S3.7: AML_TARGET specific mutated driver gene sets relative to LAML, LAML_CN and LAML_KR

K	**specific gene set**	p_1	p_2, p_3, p_4	p
3	*KIT,KRAS,NRAS*	0.011	0.337,1,0.982	0.002
4	*KIT,KRAS,NRAS,PTPN11*	<0.0001	0.172,1,0.993	<0.0001

Table S3.8: AML_TARGET specific mutated driver gene sets relative to LAML_CN

K	**specific gene set**	p_1	p_2	p
3	*FLT3,KIT,NRAS*	0.006	1	0.015
4	*FLT3,KIT,NRAS,PTPN11*	0.001	1	<0.0001
5	*FLT3,KIT,NRAS,PTPN11,KRAS*	<0.0001	1	<0.0001
6	*FLT3,KIT,NRAS,PTPN11,KRAS,WT1*	<0.0001	1	<0.0001
7	*FLT3,KIT,NRAS,PTPN11,KRAS,WT1,TET2*	<0.0001	1	<0.0001
8	*FLT3,KIT,NRAS,PTPN11,KRAS,WT1,TET2,KMT2D*	<0.0001	1	<0.0001
9	*FLT3,KIT,NRAS,PTPN11,KRAS,WT1,TET2,KMT2D,IKZF1*	<0.0001	1	<0.0001
10	*FLT3,KIT,NRAS,PTPN11,KRAS,WT1,TET2,KMT2D,IKZF1,JAK2*	<0.0001	1	<0.0001

LAML_CN has no specific mutated driver gene sets relative to AML_TARGET when $K = 2 \sim 10$.

Table S3.9: AML_TARGET specific mutated driver gene sets relative to LAML_KR

K	**specific gene set**	p_1	p_2	p
3	*KIT,KRAS,NRAS*	0.01	0.988	<0.0001
4	*KIT,KRAS,NRAS,FLT3*	0.001	0.998	<0.0001
5	*KIT,KRAS,NRAS,FLT3,IKZF1*	<0.0001	1	<0.0001
6	*KIT,KRAS,NRAS,FLT3,SOS1,PTPN11*	<0.0001	1	<0.0001
7	*KIT,KRAS,NRAS,FLT3,SOS1,PTPN11,DAZAP1*	<0.0001	1	<0.0001
8	*KIT,KRAS,NRAS,FLT3,SOS1,PTPN11,DAZAP1,IKZF1*	<0.0001	1	<0.0001
9	*KIT,KRAS,NRAS,FLT3,SOS1,PTPN11,DAZAP1,IKZF1,KLHL36*	<0.0001	1	<0.0001
10	*KIT,KRAS,NRAS,FLT3,SOS1,PTPN11,DAZAP1,IKZF1,KLHL36,CXCR4*	<0.0001	1	<0.0001

LAML_KR has no specific mutated driver gene sets relative to AML_TARGET when $K = 2 \sim 10$.

Table S3.10: LAML and AML_TARGET specific mutated driver gene sets relative to each other

**Type**	K	**Specific gene set**	p_1	p_2	p
**LAML / AML_TARGET**	3	*TP53,NPM1,RUNX1*	0.01	1	0.007
	4	*TP53,NPM1,RUNX1,WT1*	0.005	1	<0.0001
	5	*ARHGAP35,RUNX1,CEBPA,DNMT3A,MTOR*	0.009	1	0.003
	6	*TP53,ARHGAP35,ASXL1,NPM1,PHF6,WT1*	0.002	1	<0.0001
	7	*ARHGAP35,CEBPA,DNMT3A,ASXL1,PHF6,SUZ12,TNC*	0.007	1	0.005
	8	*ARHGAP35,CEBPA,DNMT3A,ASXL1,PHF6,SUZ12,TNC,MTOR*	0.003	1	0.004
	9	*ARHGAP35,CEBPA,DNMT3A,ASXL1,PHF6,SUZ12,TNC,MTOR,BRCA2*	0.005	1	0.003
	10	*ARHGEF10L,CEBPA,DNMT3A,ASXL1,PHF6,HSPG2,TNC,MTOR,SMC1A,AMER1*	0.012	1	0.009
**AML_TARGET / LAML**	3	*KIT,NRAS,PTPN11*	0.014	0.385	0.014
	4	*KIT,NRAS,PTPN11,KRAS*	0.01	0.197	0.002

Acute myeloid leukemia vs Glioblastoma multiforme

There is no common driver gene set between LAML and GBM when $K = 2 \sim 10$.

Table S3.11: Significant common driver gene set between AML_TARGET and GBM_DE

K	**common gene set**	p_1	p_2	p
9	*FLT3,KIT,KLHL36,KRAS,LRP1B,MAP2K7,NRAS,PTPN11,TET2*	<0.0001	0.027	<0.0001
10	*FLT3,KIT,KLHL36,KRAS,LRP1B,MAP2K7,NRAS,PTPN11,TET2,RAC1*	<0.0001	0.01	<0.0001

Table S3.12: LAML and GBM specific mutated driver gene sets relative to each other

**Type**	K	**Specific gene set**	p_1	p_2	p
**LAML / GBM**	3	*DNMT3A,KIT,RUNX1*	0.013	1	<0.0001
	4	*DNMT3A,KIT,RUNX1,NRAS*	0.002	1	<0.0001
	5	*DNMT3A,KIT,RUNX1,NRAS,CEBPA*	<0.0001	1	<0.0001
	6	*DNMT3A,KIT,RUNX1,NRAS,CEBPA,ARHGAP35*	<0.0001	1	<0.0001
	7	*DNMT3A,KIT,RUNX1,NRAS,CEBPA,ARHGAP35,KRAS*	<0.0001	1	<0.0001
	8	*DNMT3A,KIT,RUNX1,NRAS,CEBPA,AMER1,CBL,KRAS*	<0.0001	1	<0.0001
	9	*DNMT3A,KIT,RUNX1,NRAS,CEBPA,AMER1,CBL,KRAS,RBFOX2*	<0.0001	1	<0.0001
	10	*DNMT3A,KIT,RUNX1,NRAS,CEBPA,AMER1,CBL,KRAS,RBFOX2,BTG1*	<0.0001	1	<0.0001
**GBM / LAML**	2	*PIK3CA,PTEN*	0.004	1	0.011
	3	*PIK3CA,PTEN,IDH1*	<0.0001	1	<0.0001
	4	*PIK3CA,PTEN,IDH1,PIK3R1*	0.001	1	0.002
	5	*PIK3CA,PTEN,IDH1,PIK3R1,LRP1B*	0.001	0.942	0.002
	6	*PIK3CA,PTEN,IDH1,PIK3R1,LRP1B,NUP214*	<0.0001	0.939	<0.0001
	7	*PIK3CA,PTEN,IDH1,PIK3R1,LRP1B,NUP214,SGK1*	<0.0001	0.932	<0.0001
	8	*PIK3CA,PTEN,IDH1,PIK3R1,LPAR4,NUP214,SGK1,LRP1B*	<0.0001	0.944	<0.0001
	9	*PIK3CA,PTEN,IDH1,PIK3R1,LPAR4,NUP214,SGK1,LRP1B,H3F3A*	<0.0001	0.892	<0.0001
	10	*PIK3CA,PTEN,IDH1,PIK3R1,LPAR4,NUP214,SGK1,LRP1B,H3F3A,ZNF208*	<0.0001	0.887	<0.0001

Table S3.13: AML_TARGET and GBM_DE specific mutated driver gene sets relative to each other

**Type**	K	**Specific gene set**	p_1	p_2	p
**AML_TARGET / GBM_DE**	3	*KIT,KRAS,NRAS*	0.009	0.971	0.005
	4	*KIT,KRAS,NRAS,WT1*	0.001	0.994	<0.0001
	5	*KIT,KRAS,NRAS,WT1,JAK3*	<0.0001	1	<0.0001
	6	*KIT,KRAS,NRAS,WT1,IDH2,PTPN11*	<0.0001	1	<0.0001
	7	*KIT,KRAS,NRAS,WT1,IDH2,PTPN11,DAZAP1*	<0.0001	1	<0.0001
	8	*KIT,KRAS,NRAS,WT1,IDH2,PTPN11,DAZAP1,GATA2*	<0.0001	1	<0.0001
**GBM_DE / AML_TARGET**	2	*PTPRD,RABEP1*	0.014	1	0.012
	3	*PTPRD,RABEP1,RBM38*	0.002	1	0.003
	4	*PTPRD,RABEP1,RBM38,PDGFRB*	<0.0001	1	<0.0001
	5	*PTPRD,RABEP1,RBM38,PDGFRB,SMO*	<0.0001	1	<0.0001
	6	*PTPRD,RABEP1,RBM38,PDGFRB,RGL3,CEBPA*	<0.0001	1	<0.0001
	7	*PTPRD,RABEP1,RBM38,PDGFRB,RGL3,SMO,ID3*	<0.0001	1	<0.0001
	8	*PTPRD,RABEP1,RBM38,PDGFRB,PRF1,SMO,DCAF12L2,WDR45*	<0.0001	1	<0.0001
	9	*PTPRD,RABEP1,RBM38,PDGFRB,CHD4,ID3,DCAF12L2,WDR45,FOXA1*	<0.0001	1	<0.0001
	10	*PTPRD,RABEP1,RBM38,PDGFRB,CHD4,ID3,RGL3,SMO,WDR45,CEBPA*	<0.0001	1	<0.0001

Bone Cancer

There is no common driver gene set between BOCA_UK and BOCA_BKFZ when $K = 2 \sim 10$.

Table S3.14: BOCA_UK specific mutated driver gene sets relative to BOCA_BKFZ

K	**specific gene set**	p_1	p_2	p
2	*IDH1,RBFOX1*	<0.0001	1	<0.0001
3	*IDH1,RBFOX1,IDH2*	<0.0001	1	<0.0001
4	*IDH1,RBFOX1,IDH2,PDCD1LG2*	<0.0001	1	<0.0001
5	*IDH1,RBFOX1,IDH2,PDCD1LG2,IRS4*	<0.0001	1	<0.0001
6	*IDH1,RBFOX1,IDH2,PDCD1LG2,IRS4,POLQ*	<0.0001	1	<0.0001
7	*IDH1,RBFOX1,IDH2,PDCD1LG2,IRS4,RPS3A,DIS3*	<0.0001	1	<0.0001
8	*IDH1,RBFOX1,IDH2,PDCD1LG2,IRS4,HOXC13,LZTR1,POLQ*	<0.0001	1	<0.0001
9	*IDH1,RBFOX1,IDH2,PDCD1LG2,IRS4,HOXC13,LZTR1,DIS3,RPS3A*	<0.0001	1	<0.0001
10	*IDH1,RBFOX1,IDH2,PDCD1LG2,IRS4,HOXC13,LZTR1,DIS3,RPS3A,POLQ*	<0.0001	1	<0.0001

BOCA_BKFZ has no specific mutated driver gene sets relative to BOCA_UK when $K = 2 \sim 10$.

Medulloblastoma

There is no common driver gene set between MBL_DE and MBL_DE_K when $K = 2 \sim 10$.

There is no common driver gene set between MBL_DE and MBL_CA when $K = 2 \sim 10$.

Table S3.15: Significant common driver gene set between MBL_DE_K and MBL_CA

K	**common gene set**	p_1	p_2	p
3	*FOXA1,PPP2R1A,PTPRD*	0.034	0.013	<0.0001
4	*FOXA1,PPP2R1A,PTPRD,KLHL36*	0.005	0.002	<0.0001
5	*FOXA1,PPP2R1A,PTPRD,KLHL36,RABEP1*	<0.0001	0.014	<0.0001
6	*FOXA1,PPP2R1A,PTPRD,KLHL36,RABEP1,PRF1*	0.001	0.014	<0.0001
7	*FOXA1,PPP2R1A,PTPRD,KLHL36,RABEP1,PRF1,RBM38*	<0.0001	0.025	<0.0001
8	*FOXA1,PPP2R1A,PTPRD,KLHL36,RABEP1,PRF1,RBM38,FLCN*	<0.0001	0.017	<0.0001
9	*FOXA1,PPP2R1A,PTPRD,KLHL36,RABEP1,PRF1,RBM38,FLCN,EWSR1*	<0.0001	0.006	<0.0001
10	*FOXA1,PPP2R1A,PTPRD,KLHL36,RABEP1,PRF1,RBM38,ELF4,EWSR1,ING1*	<0.0001	0.009	<0.0001

Table S3.16: MBL_DE and MBL_DE_K specific mutated driver gene sets relative to each other

**Type**	K	**Specific gene set**	p_1	p_2	p
**MBL_DE / MBL_DE_K**	3	*DDX3X,HSP90AB1,SMARCD1*	0.012	1	0.005
	4	*DDX3X,HSP90AB1,SMARCD1,CDKN1B*	<0.0001	0.998	<0.0001
	5	*DDX3X,HSP90AB1,SMARCD1,CDKN1B,SDC4*	0.001	1	<0.0001
	6	*ELF4,KDM3B,PRDM1,RAD21,SMARCD1,ZNF680*	<0.0001	1	<0.0001
	7	*CDKN2C,ELF4,KDM3B,PRDM1,RAD21,SMARCD1,SPOP*	<0.0001	1	<0.0001
	8	*CDKN2C,ELF4,KDM3B,PRDM1,RAD21,SMARCD1,SPOP,ZNF680*	<0.0001	1	<0.0001
	9	*CDKN2C,ELF4,KDM3B,PRDM1,RAD21,SMARCD1,MDM2,TGIF1,AJUBA*	<0.0001	1	<0.0001
	10	*CDKN2C,IRAK1,KDM3B,KLF5,PEG3,SMARCD1,ZNF680,TGIF1,AJUBA,SOX21*	<0.0001	1	<0.0001
**MBL_DE_K / MBL_DE**	3	*PTPRD,RABEP1,SMO*	0.004	1	0.003
	4	*PTPRD,RABEP1,SMO,RGL3*	<0.0001	1	<0.0001
	5	*PTPRD,RABEP1,SMO,RGL3,PDGFRB*	<0.0001	1	<0.0001
	6	*PTPRD,RABEP1,SMO,RGL3,PDGFRB,RBM38*	<0.0001	1	<0.0001
	7	*PTPRD,RABEP1,SMO,RGL3,PDGFRB,RBM38,WDR45*	<0.0001	1	<0.0001
	8	*PTPRD,RABEP1,SMO,RGL3,PDGFRB,RBM38,WDR45,DCAF12L2*	<0.0001	1	<0.0001
	9	*PTPRD,RABEP1,SMO,RGL3,PDGFRB,RBM38,WDR45,DCAF12L2,KLHL36*	<0.0001	1	<0.0001
	10	*PTPRD,RABEP1,SMO,RGL3,PDGFRB,RBM38,WDR45,DCAF12L2,KLHL36,CHD4*	<0.0001	1	<0.0001

Table S3.17: MBL_DE and MBL_CA specific mutated driver gene sets relative to each other

**Type**	K	**Specific gene set**	p_1	p_2	p
**MBL_DE / MBL_CA**	2	*ERBB4,U2AF1*	0.034	1	0.032
	3	*DDX3X,HSP90AB1,SMARCD1*	0.014	0.967	0.006
	4	*DDX3X,HSP90AB1,HLA-B,NIN*	0.001	0.892	0.002
	5	*DAZAP1,KDM3B,USP9X,LATS2,SMARCD1*	0.001	1	<0.0001
	6	*DAZAP1,KDM3B,USP9X,NIN,ASXL1,HLA-B*	<0.0001	0.996	<0.0001
	7	*DAZAP1,KDM3B,USP9X,NIN,ASXL1,HLA-B,TNFRSF14*	<0.0001	0.992	<0.0001
	8	*BTK,CDKN2C,KDM3B,LATS2,SMARCD1,TNFRSF14,ZNF780A,PEG3*	<0.0001	0.973	<0.0001
	9	*BTK,CDKN2C,KDM3B,LATS2,SMARCD1,TNFRSF14,ZNF780A,MED12,TRIP11*	<0.0001	0.914	<0.0001
	10	*BTK,CDKN2C,KDM3B,LATS2,SMARCD1,TNFRSF14,ZNF780A,MED12,TRIP11,IDH2*	<0.0001	0.995	<0.0001
**MBL_CA / MBL_DE**	2	*PTPRD,RBFOX2*	0.017	0.786	0.018
	3	*PTPRD,RBFOX2,AKT3*	<0.0001	0.927	<0.0001
	4	*PTPRD,RBFOX2,AKT3,PDGFRA*	<0.0001	0.973	<0.0001
	5	*PTPRD,RBFOX2,AKT3,PDGFRA,PBRM1*	<0.0001	0.976	<0.0001
	6	*PTPRD,RBFOX2,AKT3,PDGFRA,PBRM1,FN1*	<0.0001	0.979	<0.0001
	7	*PTPRD,RBFOX2,AKT3,PDGFRA,PBRM1,FN1,STIL*	<0.0001	0.992	<0.0001
	8	*PTPRD,RBFOX2,AKT3,PDGFRA,PBRM1,FN1,STIL,CCND2*	<0.0001	0.992	<0.0001
	9	*PTPRD,RBFOX2,AKT3,PDGFRA,PBRM1,FN1,STIL,CCND2,KDM3B*	<0.0001	0.997	<0.0001
	10	*PTPRD,ABCB1,AKT3,ELF3,PBRM1,FN1,STIL,FOXD4L1,MTOR,EP300*	<0.0001	1	<0.0001

Neuroblastoma

There is no common driver gene set between NBL_BROAD and NBL_TARGET when $K = 2 \sim 10$.

Table S3.18: MBL_DE_K and MBL_CA specific mutated driver gene sets relative to each other

Type	K	Specific gene set	p_1	p_2	p
**MBL_DE_K / MBL_CA**	9	*ALK,ATRX,BCL9,FBN2,FOXA1,LRP1B,NCOR2,PIK3CA,SLC45A3*	0.049	0.968	0.037
	10	*ALK,ATRX,BCL9,FBN2,FOXA1,LRP1B,NCOR2,PIK3CA,SLC45A3,WNK4*	0.029	0.975	0.033
**MBL_CA / MBL_DE_K**	10	*AFF3,AR,ARID1B,DICER1,KIT,KMT2C,LPP,PTPRC,SIN3A,TP53*	0.039	0.988	0.048

Acute Lymphoid Leukemia

There is no common driver gene set between ALL_ST and ALL_TARGET when $K = 2 \sim 10$.

Acute myeloid leukemia vs Acute Lymphoid Leukemia

There is no common driver gene set between ALL_TARGET and AML_TARGET when $K = 2 \sim 10$.

Table S3.19: Significant common driver gene set between ALL_ST and AML_TARGET

K	**specific gene set**	p_1	p_2	p
10	*TP53,FLT3,KIT,KRAS,NF1,NRAS,PTPN11,TBL1XR1,TET2,WT1*	0.028	<0.0001	<0.0001

Table S3.20: ALL_TARGET and AML_TARGET specific mutated driver gene sets relative to each other

**Type**	K	**Specific gene set**	p_1	p_2	p
**ALL_TARGET / AML_TARGET**	9	*ATF7IP,CREBBP,ELL,EZH2,JAK2,KMT2D,PHF6,TP53,ZNF814*	0.041	1	0.032
**AML_TARGET / ALL_TARGET**	3	*FLT3,KIT,NRAS*	0.012	0.881	0.004
	4	*FLT3,KIT,NRAS,PTPN11*	0.001	0.841	<0.0001
	5	*FLT3,KIT,NRAS,TET2,WT1*	<0.0001	0.889	<0.0001
	6	*FLT3,KIT,NRAS,TET2,WT1,KRAS*	<0.0001	0.923	<0.0001
	7	*FLT3,KIT,NRAS,TET2,WT1,KRAS,SF3B1*	<0.0001	0.917	<0.0001
	8	*FLT3,KIT,NRAS,TET2,WT1,KRAS,SF3B1,IKZF1*	<0.0001	0.915	<0.0001
	9	*FLT3,KIT,NRAS,TET2,WT1,KRAS,SF3B1,IKZF1,JAK3*	<0.0001	0.924	<0.0001
	10	*FLT3,KIT,NRAS,TET2,WT1,KRAS,SF3B1,IKZF1,JAK3,ERCC3*	<0.0001	0.974	<0.0001

Table S3.21: ALL_ST specific mutated driver gene sets relative to AML_TARGET

K	**specific gene set**	p_1	p_2	p
3	*TP53,TBL1XR1,NRAS*	0.032	1	0.026
4	*TP53,TBL1XR1,NRAS,NF1*	0.01	1	0.007
5	*TP53,TBL1XR1,NRAS,NF1,PIK3R1*	0.002	1	0.003
6	*TP53,TBL1XR1,NF1,PIK3CA,PIK3R1,SETD2*	0.014	1	0.011
7	*TP53,TBL1XR1,NF1,PIK3CA,PIK3R1,SETD2,CREBBP*	0.019	1	0.025
8	*TP53,TBL1XR1,NF1,PIK3CA,PIK3R1,SETD2,CREBBP,NCOR1*	0.017	1	0.011
9	*TP53,TBL1XR1,NF1,PIK3CA,PIK3R1,SETD2,CREBBP,NCOR1,EZH2*	0.002	0.994	0.002
10	*FAT1,MED12,BIRC6,PIK3CA,PIK3R1,SETD2,CREBBP,NCOR1,ETV6,PDGFRB*	0.004	0.981	0.005

AML_TARGET has no specific mutated driver gene sets relative to ALL_ST when $K = 2 \sim 10$.

Glioblastoma multiforme vs Medulloblastoma vs Neuroblastoma vs Pilocytic Astrocytoma

Table S3.22: Significant common driver gene set between GBM_DE and MBL_DE_K

K	**common gene set**	p_1	p_2	p
2	*PTPRD,RABEP1*	0.017	0.024	<0.0001
3	*PTPRD,RABEP1,RBM38*	<0.0001	0.001	<0.0001
4	*PTPRD,RABEP1,RBM38,PDGFRB*	<0.0001	<0.0001	<0.0001
5	*PTPRD,RABEP1,RBM38,PDGFRB,RGL3*	<0.0001	<0.0001	<0.0001
6	*PTPRD,RABEP1,RBM38,PDGFRB,WDR45,FOXA1*	<0.0001	<0.0001	<0.0001
7	*PTPRD,RABEP1,RBM38,PDGFRB,CEBPA,CHD4,DCAF12L2*	<0.0001	<0.0001	<0.0001
8	*PTPRD,RABEP1,RBM38,PDGFRB,KLHL36,PRF1,SMO,WDR45*	<0.0001	<0.0001	<0.0001
9	*PTPRD,RABEP1,RBM38,PDGFRB,DCAF12L2,ID3,FOXA1,WDR45,PRF1*	<0.0001	<0.0001	<0.0001
10	*PTPRD,RABEP1,RBM38,PDGFRB,DCAF12L2,ID3,SMO,CEBPA,CHD4,RGL3*	<0.0001	<0.0001	<0.0001

There is no common driver gene set between GBM_DE and PA_DE when $K = 2 \sim 10$.

There is no common driver gene set between GBM_DE and NBL_TARGET when $K = 2 \sim 10$.

There is no common driver gene set between GBM_DE and NBL_BROAD when $K = 2 \sim 10$.

There is no common driver gene set between PA_DE and NBL_TARGET when $K = 2 \sim 10$.

There is no common driver gene set between PA_DE and NBL_BROAD when $K = 2 \sim 10$.

There is no common driver gene set between PA_DE and MBL_DE_K when $K = 2 \sim 10$.

There is no common driver gene set between PA_DE and MBL_CA when $K = 2 \sim 10$.

There is no common driver gene set between NBL_TARGET and MBL_DE_K when $K = 2 \sim 10$.

Table S3.23: Significant common driver gene set between GBM_DE and MBL_CA

K	**common gene set**	p_1	p_2	p
3	*FOXA1,PPP2R1A,PTPRD*	0.039	0.021	<0.0001
4	*FOXA1,PPP2R1A,PTPRD,KLHL36*	0.011	0.004	<0.0001
5	*FOXA1,PPP2R1A,PTPRD,KLHL36,RABEP1*	0.001	0.018	<0.0001
6	*FOXA1,PPP2R1A,PTPRD,KLHL36,RABEP1,PRF1*	<0.0001	0.014	<0.0001
7	*FOXA1,PPP2R1A,PTPRD,KLHL36,RABEP1,PRF1,RBM38*	<0.0001	0.041	<0.0001
8	*FOXA1,PPP2R1A,PTPRD,KLHL36,RABEP1,PRF1,RBM38,FLCN*	<0.0001	0.012	<0.0001
9	*FOXA1,PPP2R1A,PTPRD,KLHL36,RABEP1,PRF1,RBM38,ELF4,EWSR1*	<0.0001	0.021	<0.0001
10	*FOXA1,PPP2R1A,PTPRD,KLHL36,RABEP1,PRF1,RBM38,ELF4,EWSR1,FLCN*	<0.0001	0.002	<0.0001

There is no common driver gene set between NBL_TARGET and MBL_CA when $K = 2 \sim 10$.

There is no common driver gene set between NBL_BROAD and MBL_DE_K when $K = 2 \sim 10$.

There is no common driver gene set between NBL_BROAD and MBL_CA when $K = 2 \sim 10$.

There is no specific driver gene set between GBM_DE and MBL_DE_K relative to each other when $K = 2 \sim 10$.

Table S3.24: GBM_DE and MBL_CA specific mutated driver gene sets relative to each other

**Type**	K	**Specific gene set**	p_1	p_2	p
**GBM_DE / MBL_CA**	2	*KLHL6,LRP1B*	0.046	0.81	0.033
	3	*CASP8,LRP1B,PDGFRB*	0.003	1	<0.0001
	4	*CASP8,LRP1B,PDGFRB,RAC1*	0.001	1	<0.0001
	5	*CASP8,LRP1B,PDGFRB,RAC1,SMO*	<0.0001	0.993	<0.0001
	6	*CASP8,LRP1B,PDGFRB,RAC1,SMO,CCR7*	<0.0001	0.999	<0.0001
	7	*CASP8,LRP1B,PDGFRB,RAC1,SMO,CCR7,PIM1*	<0.0001	0.991	<0.0001
	8	*CASP8,LRP1B,PDGFRB,RAC1,SMO,CCR7,PIM1,HOXC13*	<0.0001	0.997	<0.0001
	9	*CASP8,LRP1B,PDGFRB,RAC1,SMO,CCR7,PIM1,HOXC13,HSP90AB1*	<0.0001	0.996	<0.0001
	10	*CASP8,LRP1B,PDGFRB,RAC1,SMO,CCR7,PIM1,HOXC13,HSP90AB1,PPT2*	<0.0001	0.999	<0.0001
**MBL_CA / GBM_DE**	2	*PTPRD,RBFOX2*	0.01	0.985	0.001
	3	*CLIP1,NUMA1,PTCH1*	0.007	0.989	0.001
	4	*CLIP1,NUMA1,PTCH1,STK11*	0.004	0.998	<0.0001
	5	*CLIP1,NUMA1,PTCH1,STK11,CLTC*	<0.0001	0.999	<0.0001
	6	*CLIP1,NUMA1,PTCH1,STK11,CLTC,DDB2*	0.001	0.999	<0.0001
	7	*CLIP1,NUMA1,PTCH1,STK11,CLTC,DDB2,XPC*	<0.0001	1	<0.0001
	8	*CLIP1,NUMA1,PTCH1,STK11,CLTC,DDB2,XPC,LZTR1*	<0.0001	0.999	<0.0001
	9	*CLIP1,NUMA1,PTCH1,STK11,CLTC,DDB2,XPC,LZTR1,ELF4*	<0.0001	1	<0.0001
	10	*CLIP1,NUMA1,PTCH1,STK11,CLTC,DDB2,XPC,LZTR1,ETV4,CLTCL1*	<0.0001	1	<0.0001

Table S3.25: GBM_DE specific mutated driver gene sets relative to NBL_TARGET and NBL_BROAD

K	**specific gene set**	p_1	p_2, p_3	p
2	*PTPRD,RABEP1*	0.014	1,1	0.026
3	*PTPRD,RABEP1,RBM38*	0.002	1,1	<0.0001
4	*PTPRD,RABEP1,RBM38,PDGFRB*	<0.0001	1,1	<0.0001
5	*PTPRD,RABEP1,RBM38,PDGFRB,SMO*	<0.0001	1,1	<0.0001
6	*PTPRD,RABEP1,RBM38,PDGFRB,SMO,CEBPA*	<0.0001	1,1	<0.0001
7	*PTPRD,RABEP1,RBM38,PDGFRB,DCAF12L2,ID3,KLHL36*	<0.0001	1,1	<0.0001
8	*PTPRD,RABEP1,RBM38,PDGFRB,DCAF12L2,ID3,KLHL36,CHD4*	<0.0001	1,1	<0.0001
9	*PTPRD,RABEP1,RBM38,PDGFRB,DCAF12L2,ID3,CEBPA,CHD4,WDR45*	<0.0001	1,1	<0.0001
10	*PTPRD,RABEP1,RBM38,PDGFRB,DCAF12L2,ID3,KLHL36,CHD4,WDR45,SMO*	<0.0001	1,1	<0.0001

NBL_BROAD and NBL_TARGET has no specific mutated driver gene sets relative to GBM_DE when $K = 2 \sim 10$.

Table S3.26: GBM_DE specific mutated driver gene sets relative to PA_DE

K	**specific gene set**	p_1	p_2	p
2	*PTPRD,RABEP1*	0.017	1	0.025
3	*PTPRD,RABEP1,RBM38*	<0.0001	1	0.003
4	*PTPRD,RABEP1,RBM38,PDGFRB*	0.001	1	<0.0001
5	*PTPRD,RABEP1,RBM38,PDGFRB,FOXA1*	<0.0001	1	<0.0001
6	*PTPRD,RABEP1,RBM38,PDGFRB,FOXA1,KLHL36*	<0.0001	1	<0.0001
7	*PTPRD,RABEP1,RBM38,PDGFRB,FOXA1,KLHL36,RGL3*	<0.0001	1	<0.0001
8	*PTPRD,RABEP1,RBM38,PDGFRB,FOXA1,CHD4,RGL3,WDR45*	<0.0001	1	<0.0001
9	*PTPRD,RABEP1,RBM38,PDGFRB,FOXA1,CHD4,RGL3,DCAF12L2,KLHL36*	<0.0001	1	<0.0001
10	*PTPRD,RABEP1,RBM38,PDGFRB,FOXA1,CHD4,RGL3,DCAF12L2,KLHL36,ID3*	<0.0001	1	<0.0001

PA_DE has no specific mutated driver gene sets relative to GBM_DE when $K = 2 \sim 10$.

Table S3.27: NBL_BROAD specific mutated driver gene sets relative to PA_DE

K	**specific gene set**	p_1	p_2	p
5	*ALK,FBN2,LRP1B,NCOR2,PTPN11*	0.033	1	0.044
6	*ALK,FBN2,LRP1B,MYCN,PTPN11,FLT4*	<0.0001	1	<0.0001
7	*ALK,FBN2,LRP1B,MYCN,PTPN11,FLT4,ATRX*	0.032	1	0.036
8	*ALK,FBN2,LRP1B,MYCN,PTPN11,FLT4,ATRX,ATM*	0.014	1	0.02
9	*ALK,FBN2,LRP1B,MYCN,PTPN11,FLT4,ATRX,ATM,IRS4*	0.011	1	0.009
10	*ALK,FBN2,LRP1B,MYCN,PTPN11,FLT4,ATRX,ATM,IRS4,ZNF208*	0.007	1	0.007

PA_DE has no specific mutated driver gene sets relative to NBL_BROAD when $K = 2 \sim 10$.

Table S3.28: MBL_DE_K and MBL_CA specific mutated driver gene sets relative to NBL_TARGET and NBL_BROAD

K	**specific gene set**	p_1, p_2	p_3, p_4	p
4	*KLHL36,PPP2R1A,PTPRD,ZNF721*	0.003,0.015	1,1	<0.0001
5	*KLHL36,PPP2R1A,PTPRD,ZNF721,RBM38*	<0.0001,0.04	1,1	<0.0001
7	*KLHL36,PPP2R1A,PTPRD,ZNF721,RBM38,GNA11,PRF1*	<0.0001	1,1	<0.0001
8	*KLHL36,PPP2R1A,PTPRD,ZNF721,RBM38,GNA11,PRF1,EWSR1*	<0.0001,0.02	1,1	<0.0001
9	*KLHL36,PPP2R1A,PTPRD,ZNF721,RBM38,GNA11,PRF1,FLCN,ING1*	<0.0001,0.007	1,1	<0.0001
10	*KLHL36,PPP2R1A,PTPRD,ZNF721,RBM38,GNA11,PRF1,FLCN,U2AF2,ID3*	<0.0001,0.03	1,1	<0.0001

NBL_BROAD and NBL_TARGET has no specific mutated driver gene sets relative to MBL_DE_K and MBL_CA when $K = 2 \sim 10$.

Table S3.29: MBL_DE_K and MBL_CA specific mutated driver gene sets relative to PA_DE

K	**specific gene set**	p_1, p_2	p_3	p
3	*PPP2R1A,PTPRD,FOXA1*	0.032,0.013	1	0.001
4	*PPP2R1A,PTPRD,KLHL36,ZNF721*	0.002,0.02	1	<0.0001
5	*PPP2R1A,PTPRD,KLHL36,FOXA1,RABEP1*	<0.0001,0.015	1	<0.0001
6	*PPP2R1A,PTPRD,KLHL36,FOXA1,RABEP1,RBM38*	<0.0001,0.041	1	<0.0001
7	*PPP2R1A,PTPRD,KLHL36,FOXA1,RABEP1,RBM38,PRF1*	<0.0001,0.041	1	<0.0001
8	*PPP2R1A,PTPRD,KLHL36,FOXA1,RABEP1,RBM38,PRF1,FLCN*	<0.0001,0.01	1	<0.0001
9	*PPP2R1A,PTPRD,KLHL36,FOXA1,RABEP1,RBM38,PRF1,EWSR1,RGL3*	<0.0001,0.044	1	<0.0001
10	*PPP2R1A,PTPRD,KLHL36,FOXA1,RABEP1,RBM38,PRF1,EWSR1,RGL3,FLCN*	<0.0001,0.016	1	<0.0001

PA_DE has no specific mutated driver gene sets relative to MBL_DE_K and MBL_CA when $K = 2 \sim 10$.

4 Tables: Environmental factors may promote pathogenicity by pathway

Smoke

Lung adenocarcinoma

Table S4.1: LUAD S and N_S specific mutated driver gene sets relative to each other

**Type**	K	**Specific gene set**	p_1	p_2	p
**LUAD S / N_S**	2	*TP53,KRAS*	0.001	0.954	0.004
	3	*TP53,KRAS,CTNNB1*	0.012	0.977	0.003
	4	*KEAP1,LRP1B,NBEA,ARHGAP5*	0.015	0.978	<0.0001
	5	*KEAP1,LRP1B,NBEA,NKX2-1,ARHGAP5*	0.001	0.988	0.002
	6	*KEAP1,LRP1B,NBEA,NKX2-1,NPM1,SETDB1*	<0.0001	0.87	0.001
	7	*KEAP1,LRP1B,DNMT3A,NKX2-1,NPM1,SETDB1,KDM5A*	<0.0001	0.997	0.001
	8	*KEAP1,CMTR2,NBEA,FLT3,PLCB4,SMC1A,SOX9,STK11*	<0.0001	0.937	<0.0001
	9	*KEAP1,CMTR2,NBEA,FLT3,PLCB4,SMC1A,SOX9,STK11,BAP1*	<0.0001	0.998	<0.0001
	10	*ARID1A,BTK,NBEA,DCSTAMP,EBF1,FANCD2,KDM5C,STK11,RUNX1T1,TNC*	<0.0001	0.992	<0.0001
**LUAD N_S / S**	3	*BCL6,EGFR,MET*	0.021	0.473	<0.0001
	4	*CYSLTR2,EGFR,IDH1,AXIN1*	0.012	0.95	0.001
	5	*CYSLTR2,EGFR,IDH1,AXIN1,CASP8*	0.005	1	0.001
	6	*CYSLTR2,EGFR,IDH1,AXIN1,CASP8,SDC4*	0.007	1	0.001
	7	*CYSLTR2,EGFR,IDH1,AXIN1,CASP8,SDC4,SOX21*	0.004	1	0.011
	8	*CYSLTR2,EGFR,IDH1,SDC4,CASP8,TBL1XR1,PAK2,POLD1*	0.001	1	<0.0001
	9	*CYSLTR2,EGFR,IDH1,SDC4,CASP8,TBL1XR1,PAK2,MLLT1,BCL2*	<0.0001	1	<0.0001
	10	*CYSLTR2,EGFR,IDH1,SDC4,CASP8,BCL2,CXCR4,PAK2,MLLT1,SOHLH2*	<0.0001	1	<0.0001

S: Smokers; N_S: Nonsmokers.

Head and neck squamous cell carcinoma

Table S4.2: HNSC S and N_S specific mutated driver gene sets relative to each other

**Type**	K	**Specific gene set**	p_1	p_2	p
**HNSC S / N_S**	4	*PIK3CA,RUNX1T1,TRRAP,EP300*	0.021	0.963	0.012
	5	*PIK3CA,RUNX1T1,TRRAP,EP300,XPO1*	0.001	0.966	0.001
	6	*PIK3CA,RUNX1T1,TRRAP,EP300,XPO1,NKTR*	0.001	0.912	0.004
	7	*PIK3CA,RUNX1T1,TRRAP,EP300,XPO1,NKTR,COL1A1*	<0.0001	0.999	0.001
	8	*PIK3CA,RUNX1T1,TRRAP,FBXW7,LPAR4,TLL1,COL1A1,PBRM1*	0.001	1	<0.0001
	9	*PIK3CA,RUNX1T1,TRRAP,FBXW7,LPAR4,TLL1,COL1A1,PBRM1,NKTR*	<0.0001	1	<0.0001
	10	*PIK3CA,RUNX1T1,TRRAP,FBXW7,LPAR4,TLL1,COL1A1,PBRM1,NKTR,KDM3B*	0.001	0.922	<0.0001
**HNSC N_S / S**	3	*CACNA1D,CDKN2A,GRIN2A*	<0.0001	0.955	<0.0001
	4	*CACNA1D,CDKN2A,GRIN2A,AXIN1*	<0.0001	0.95	0.001
	5	*CACNA1D,CDKN2A,GRIN2A,AXIN1,P2RY8*	<0.0001	1	0.001
	6	*CACNA1D,CDKN2A,GRIN2A,AXIN1,P2RY8,ASXL1*	<0.0001	1	<0.0001
	7	*CASP8,CR1,ERCC2,GRIN2A,JAK1,KMT2A,WNK4*	<0.0001	1	<0.0001
	8	*CASP8,CR1,ERCC2,GRIN2A,JAK1,KMT2A,WNK4,AXIN1*	<0.0001	1	<0.0001
	9	*CACNA1D,APC,BRAF,CR1,ERBB2,JAK2,NCOR1,RASA1,ZFP36L1*	<0.0001	1	<0.0001
	10	*CACNA1D,APC,BRAF,CR1,ERBB2,JAK2,NCOR1,RASA1,ZFP36L1,ELN*	<0.0001	1	<0.0001

Bladder cancer

Table S4.3: BLCA S and N_S specific mutated driver gene sets relative to each other

**Type**	K	**Specific gene set**	p_1	p_2	p
**BLCA S / N_S**	2	*TP53,FGFR3*	<0.0001	0.091	0.003
	3	*RB1,ARID1A,STAG2*	0.018	0.922	0.005
	4	*RB1,ELF3,FBN2,KRAS*	<0.0001	0.948	<0.0001
	5	*RB1,ELF3,FBN2,NFE2L2,BIRC6*	<0.0001	0.933	<0.0001
	6	*RB1,ELF3,FBN2,KRAS,NFE2L2,SUZ12*	<0.0001	0.848	<0.0001
	7	*RB1,ELF3,DCAF12L2,KRAS,PTPRK,SUZ12,UBR5*	<0.0001	0.911	<0.0001
	8	*RB1,ELF3,FBN2,KRAS,NFE2L2,SUZ12,U2AF2,PRF1*	<0.0001	0.937	<0.0001
	9	*RB1,ELF3,PRF1,KRAS,PTPRK,SUZ12,U2AF2,UBR5,FLCN*	<0.0001	1	<0.0001
	10	*ABCB1,ELF3,FLCN,KRAS,PRF1,PTPRK,RB1,SUZ12,U2AF2,UBR5*	<0.0001	1	<0.0001
**BLCA N_S / S**	2	*TP53,CDKN1A*	0.002	0.366	0.011
	3	*IRS4,KDM6A,KMT2D*	<0.0001	0.677	<0.0001
	4	*FN1,PIK3CA,PTPRB,RHOA*	0.005	0.95	0.002
	5	*DNMT3A,PSIP1,PTPRB,RASA1,TRIM24*	0.013	1	0.001
	6	*DNMT3A,PSIP1,PTPRB,RASA1,TRIM24,STAT6*	0.011	1	<0.0001
	7	*IDH1,IRS4,PSIP1,TRRAP,RAP1GDS1,TRIM24,STAT6*	0.001	1	<0.0001
	8	*NOTCH2,PSIP1,PTPRB,RAP1GDS1,RASA1,STAT6,TRIM24,WNK4*	<0.0001	1	<0.0001
	9	*NOTCH2,PSIP1,PTPRB,RAP1GDS1,RASA1,STAT6,TRIM24,WNK4,SPOP*	<0.0001	1	<0.0001
	10	*NOTCH2,PSIP1,PTPRB,RAP1GDS1,RASA1,STAT6,TRIM24,WNK4,SPOP,PAK2*	<0.0001	1	<0.0001

Alcohol

Head and neck squamous cell carcinoma

Table S4.4: HNSC A and N_A specific mutated driver gene sets relative to each other

**Type**	K	**Specific gene set**	p_1	p_2	p
**HNSC A / N_A**	4	*CDKN2A,LRP1B,NSD1,SMAD4*	0.048	0.986	0.002
	5	*CDKN2A,NOTCH1,PTPRD,SETBP1,ZNF521*	0.025	0.993	0.025
	6	*CDKN2A,NOTCH1,PTPRD,SETBP1,ZNF521,CASZ1*	0.011	0.994	0.002
	7	*CDKN2A,NOTCH1,PTPRD,SETBP1,ZNF521,CASZ1,CTCF*	0.004	0.995	<0.0001
	8	*CDKN2A,NOTCH1,PTPRD,SETBP1,ZNF521,CASZ1,CTCF,AFF1*	<0.0001	1	<0.0001
	9	*CDKN2A,NOTCH1,PTPRD,SETBP1,ZNF521,CASZ1,CTCF,AFF1,BRCA1*	<0.0001	1	<0.0001
	10	*CDKN2A,NOTCH1,PTPRD,SETBP1,ZNF521,CASZ1,CTCF,AFF1,BRCA1,INO80*	<0.0001	1	<0.0001
**HNSC N_A / A**	4	*CASP8,MAPK1,NRP1,ZNF626*	0.048	0.972	0.012
	5	*CASP8,MAPK1,NRP1,ZNF626,STAT3*	0.009	0.993	0.001
	6	*CASP8,MAPK1,NRP1,ZNF626,STAT3,ETV5*	0.004	1	<0.0001
	7	*CASP8,MAPK1,NRP1,ZNF626,STAT3,ETV5,FH*	0.001	0.997	<0.0001
	8	*CASP8,MAPK1,NRP1,ZNF626,STAT3,ETV5,EXT2,FH*	<0.0001	0.999	<0.0001
	9	*CASP8,MAPK1,NRP1,ZNF626,STAT3,ETV5,EXT2,FH,RUNX1*	0.001	1	<0.0001
	10	*CASP8,MAPK1,NRP1,ZNF626,STAT3,ETV5,EXT2,FH,RUNX1,FBLN1*	<0.0001	1	<0.0001

A: Drinkers; N_A: Nondrinkers.

Esophageal carcinoma

Table S4.5: ESCA A and N_A specific mutated driver gene sets relative to each other

**Type**	K	**Specific gene set**	p_1	p_2	p
**ESCA A / N_A**	2	*TP53,PTPRD*	<0.0001	0.097	<0.0001
	3	*TP53,LDB1,SOX9*	0.001	0.983	0.004
	4	*TP53,LDB1,CYLD,PRR14*	0.026	0.983	0.001
	5	*TP53,LDB1,CYLD,PRR14,ZBTB16*	0.021	0.933	0.003
	6	*APC,NBEA,NFE2L2,NOTCH1,SMAD4,MAP3K1*	0.001	0.997	0.001
	7	*APC,NBEA,NFE2L2,NOTCH1,SMAD4,MAP3K1,GNAS*	0.003	1	<0.0001
	8	*APC,NBEA,NFE2L2,NOTCH1,SMAD4,ATF7IP,FAT4,HERC2*	<0.0001	0.921	<0.0001
	9	*APC,NBEA,NFE2L2,NOTCH1,SMAD4,ATF7IP,FAT4,HERC2,ATM*	<0.0001	1	<0.0001
	10	*APC,NBEA,NFE2L2,NOTCH1,SMAD4,ATF7IP,FAT4,HERC2,ATM,CLTC*	<0.0001	1	<0.0001
**ESCA N_A / A**	3	*PTPRD,CDKN2A,FAT3*	0.001	0.761	0.001
	4	*PTPRD,ATG7,FAT3,LZTR1*	0.003	0.853	0.001
	5	*DCAF12L2,NIPBL,PTPN13,SATB1,TNC*	0.001	0.911	0.001
	6	*DCAF12L2,NIPBL,PTPN13,SATB1,TNC,NOTCH2*	0.001	0.923	<0.0001
	7	*DCAF12L2,NIPBL,PTPN13,SATB1,NOTCH2,FANCD2,KIT*	<0.0001	0.975	<0.0001
	8	*DCAF12L2,NIPBL,ATG7,CASZ1,CUL3,NOTCH2,FBXW7,KIT*	<0.0001	0.997	<0.0001
	9	*DCAF12L2,NIPBL,ATG7,CASZ1,NOTCH2,CUL3,FBXW7,KIT,CBFA2T3*	<0.0001	0.998	<0.0001
	10	*DCAF12L2,NIPBL,ATG7,CASZ1,NOTCH2,CUL3,FBXW7,KIT,CBFA2T3,ZNF148*	<0.0001	0.994	<0.0001

Pancreatic adenocarcinoma

Table S4.6: PAAD A specific mutated driver gene sets relative to PAAD N_A

K	**specific gene set**	p_1	p_2	p
2	*KRAS,GNAS*	0.011	0.976	0.021
3	*KRAS,GNAS,MYO5A*	<0.0001	0.92	0.001
4	*KRAS,GNAS,MYO5A,MEN1*	0.001	0.988	<0.0001
5	*KRAS,GNAS,MYO5A,MEN1,FOXA2*	<0.0001	0.999	<0.0001
6	*KRAS,GNAS,MYO5A,FOXA2,CD79B,SPEN*	<0.0001	1	<0.0001
7	*KRAS,FHIT,MYO5A,FOXA2,CD79B,SPEN,PTEN*	<0.0001	1	<0.0001
8	*KRAS,FHIT,MYO5A,FOXA2,CD79B,FLT4,PTEN,MEN1*	<0.0001	1	<0.0001
9	*KRAS,FHIT,MYO5A,FOXA2,CD79B,FLT4,PTEN,EML4,CRNKL1*	<0.0001	1	<0.0001
10	*KRAS,FHIT,MYO5A,BAP1,CD79B,FLT4,PTEN,MEN1,NTRK3,CBL*	<0.0001	1	<0.0001

PAAD N_A has no specific mutated driver gene sets relative to PAAD A when $K = 2 \sim 10$.

BMI

Liver hepatocellular carcinoma

Table S4.7: LIHC O and N_O specific mutated driver gene sets relative to each other

Type	K	Specific gene set	p_1	p_2	p
LIHC O / N_O	4	*APC,ATM,ALB,IRS4*	0.023	0.876	0.012
	5	*APC,ATM,ALB,IRS4,CHD4*	0.016	0.896	0.002
	6	*APC,ATM,ACVR2A,CDKN1A,KEAP1,CHD4*	0.002	0.993	<0.0001
	7	*APC,ATM,ACVR2A,CDKN1A,KEAP1,CHD4,WRN*	<0.0001	0.996	<0.0001
	8	*APC,ATM,ACVR2A,CDKN1A,KEAP1,NIPBL,WRN,USP6*	<0.0001	0.997	0.001
	9	*APC,ATM,ACVR2A,CDKN1A,KEAP1,CHD4,HIP1,WRN,SGK1*	<0.0001	1	0.002
	10	*APC,ATM,ACVR2A,CDKN1A,KEAP1,HIP1,NIPBL,WRN,SGK1,USP6*	<0.0001	1	<0.0001
LIHC N_O / O	3	*TP53,AXIN1,CUX1*	0.021	0.776	0.026
	4	*BRD7,HERC2,LRP1B,SETD2*	0.011	0.821	0.001
	5	*BRD7,HERC2,LRP1B,SETD2,CDKN2A*	0.001	0.892	0.002
	6	*BRD7,EP300,RASA1,RB1,SETD2,SMARCA1*	0.011	0.991	0.021
	7	*EP300,HERC2,HSPG2,JAK1,LRIG3,RB1,SMARCA1*	0.001	0.993	0.001
	8	*EP300,HERC2,HSPG2,JAK1,LRIG3,RB1,SMARCA1,PIK3CB*	0.003	0.989	0.002
	9	*EP300,HERC2,HSPG2,JAK1,LRIG3,RB1,SMARCA1,PIK3CB,NKTR*	0.001	0.999	<0.0001
	10	*BRD7,EP300,ARHGEF12,BCL11A,RB1,SMARCA1,PIK3R1,NKTR,RASA1,SETD2*	<0.0001	0.978	0.011

O: obese; N_O: Nonobese

Colorectal Cancer

Table S4.8: COADREAD O and N_O specific mutated driver gene sets relative to each other

**Type**	K	**Specific gene set**	p_1	p_2	p
**COADREAD O / N_O**	2	*TP53,WNK2*	0.001	0.892	0.002
	3	*TP53,PLCG1,WNK2*	0.011	0.991	0.021
	4	*TP53,ATR,KLHL36,USP8*	0.001	0.993	0.001
	5	*TP53,ATR,KLHL36,USP8,PLAG1*	<0.0001	0.993	<0.0001
	6	*TP53,ATR,KLHL36,USP8,CDKN1B,ZNF680*	<0.0001	0.997	<0.0001
	7	*TP53,ATR,KDM5C,PLAG1,USP8,CHEK2,DROSHA*	<0.0001	1	<0.0001
	8	*TP53,ATR,CDKN2C,CHEK2,IKZF1,KDM5C,USP8,PLAG1*	<0.0001	1	<0.0001
	9	*TP53,ATR,CDKN2C,DROSHA,GATA1,KDM5C,USP8,CDKN1B,CCR7*	<0.0001	1	<0.0001
	10	*TP53,ATR,CDKN2C,GATA1,IKZF1,KDM5C,USP8,SIX1,CDKN1B,CCR7*	<0.0001	1	<0.0001
**COADREAD N_O / O**	5	*EPHA3,PRRX1,FOXP1,PML,RPL10*	0.04	0.991	0.001
	8	*EPHA3,PRRX1,ELL,MAP2K1,MAX,MYD88,PLCG1,STIL*	0.007	1	<0.0001

Skin Cutaneous Melanoma

Table S4.9: SKCM O and N_O specific mutated driver gene sets relative to each other

Type	K	Specific gene set	p_1	p_2	p
SKCM O / N_O	3	*BRAF,KIT,NRAS*	0.031	0.812	0.022
	4	*ERBB3,LRP1B,MALT1,PTPN11*	0.011	0.891	0.012
	5	*ERBB3,LRP1B,MALT1,PTPN11,BRD7*	0.01	0.762	0.002
	6	*ERBB3,LRP1B,MALT1,BRD7,DDB2,FGFR3*	<0.0001	0.921	<0.0001
	7	*BTK,DDX3X,FUBP1,IKZF1,MAP2,PTPRB,ZBTB20*	0.001	0.992	<0.0001
	8	*BTK,DDX3X,FUBP1,IKZF1,MAP2,PTPRB,ZBTB20,EXT2*	0.002	0.999	<0.0001
	9	*BTK,DDX3X,FUBP1,IKZF1,MAP2,PTPRB,ZBTB20,LDB1,SMC1A*	<0.0001	1	<0.0001
	10	*CARS,DDX3X,EXT2,IKZF1,MAP2K1,NOTCH2,ZBTB20,NXF1,PTEN,SPOP*	<0.0001	1	<0.0001
SKCM N_O / O	8	*ASXL1,BCOR,IL6ST,IRS4,SUZ12,PIM1,TRAF3,SRSF2*	0.002	0.99	0.021
	9	*CDH1,ELL,IL6ST,MEN1,PAK2,PIM1,RUNX1,SUZ12,TRAF3*	0.001	0.899	0.001
	10	*CDH1,ELL,IL6ST,MEN1,PAK2,PIM1,RUNX1,SUZ12,SRSF2,DROSHA*	0.002	0.923	0.003
