# Supplementary material for: Multi-context modeling of driver pathways reveals common and specific mechanisms across 23 cancer types
Source: PLoS Comput Biol. 2025 Aug 6;21(8):e1013349. doi: 10.1371/journal.pcbi.1013349 (PMC12349879; doi:10.1371/journal.pcbi.1013349)
Supplement: Attachment — Submitted filename: Response to Reviewers.docx [file pcbi.1013349.s009.docx]

**Responses to the Reviewers’ comments:**

We would like to thank the editor and all reviewers for their insightful, perceptive, and valuable comments on this manuscript. We wholeheartedly embrace all of the suggestions put forth by the reviewers, and we have carefully incorporated these suggestions by implementing relevant revisions in both the main text and Supporting Information. These comments and suggestions have helped us to improve our manuscript in many aspects. The reviewer comments are numbered, and presented below in blue italics. Our corresponding responses are provided in standard font.

************
*Reviewer #1 (Comments to the Author):

In this manuscript, the authors conduct a comprehensive analysis of multiple cancer omics data by focusing on the identificaiton of cancer driver genes and pathways. Overall, the manuscript is informative. However, the current manuscript should be revised before acceptance for publication.*

**Response:**

We express our sincere gratitude to the reviewer for the favorable assessment and thought-provoking suggestions regarding our manuscript.

*There is a few aspects however that need clarifications:

1. The current title and abstract are too long, preventing readers from comprehending the key points. The authors should rewrite the title and abstract to make sure that the most novel findings are clearly presented.*

**Response:**

We sincerely appreciate your constructive suggestion to improve the clarity of our manuscript. As recommended, we have thoroughly revised the title and abstract to concisely highlight the novel findings while reducing redundancy.

(1) Title Revision:

The original title has been shortened and refocused to emphasize the multi-context modeling approach and key outcome (common/specific mechanisms). The new title:

"Multi-context modeling of driver pathways reveals common and specific mechanisms across 23 cancer types".

(2) Abstract Revision:

Key modifications include:

(a) Shortens the text to ~ 150 words while retaining all critical information.

(b) Directly states the gap ("context-specific patterns across populations remain poorly understood") and our approach (stratified analysis of 23 cancers via EntCDP / ModSDP).

(c) Clearly separates the four perspectives (region, tumor type, age, risk factors) with specific examples (e.g., PI3K-Akt in Chinese vs. GPCR in American with bladder cancer; NOTCH-alcohol link).

(d) Emphasizes the translational impact—prioritizing “context-aware therapeutic targets.”

The modified abstract is:

Discovery of cancer driver pathways is essential for targeted therapies, since pathways govern tumor progression and treatment resistance. However, their context-specific patterns across populations remain poorly understood. Leveraging pan-cancer genomic data, we apply our two models, EntCDP and ModSDP, to perform stratified analyses from four perspectives: region, tumor type, age group, and risk factor. Our results reveal the regional biases in perturbed pathway, such as PI3K-Akt in Chinese and GPCR in American patients with bladder cancer. Subtype comparisons highlight mTOR signaling in lung adenocarcinoma versus FoxO signaling in lung squamous cell carcinoma. Pediatric-adult comparisons emphasize the enrichment of Ras and PAK signaling in pediatric acute myeloid leukemia and glioblastoma, respectively. Risk factor associations further link NOTCH-mediated pathways to alcohol and CDKN-regulated signals to obesity-related cancers. Our findings demonstrate the utility of stratified driver pathway analysis in uncovering common and specific mechanisms, which can help prioritize context-aware therapeutic target.

We appreciate your suggestion, which significantly improved the manuscript’s accessibility. We hope the revised content meets your expectations.

*2. Findings related to regions, ages, and environmental risky factors are comprehensive, but hard to be validated experimentally. The authors should prioritize the most important and novel points from these comprehensive findings. It is best to form a testable hypothesis.*

**Response:**

We appreciate the reviewer’s valuable comments on the comprehensiveness of our findings and the suggestion to emphasize testable hypotheses. We acknowledge that experimental validation remains challenging. In response, we have made several key revisions:

(1) We emphasized novel and potentially testable findings, especially those with implications for future experimental validation. For example:

(a) In subsection ‘Targeting novel driver genes in region-specific signaling pathways ’, we emphasize the new discovery of region-specific genes: ‘Apart from these genes in specific pathways, some light gray genes in Fig \ref{fig2}D, such as \emph{ARAF}, \emph{ESRRA} and \emph{PTMA} (from the China cohort), as well as \emph{KDM6A}, \emph{PABPC1} and \emph{TRIM49C} (from the American cohort), are predicted by ModSDP as region-specific drivers of bladder cancer and warrant further experimental validation.’ (Line 202)

(b) In subsection ‘Signal in esophageal and gastric carcinoma’, we emphasize the new discovery of \emph{TP53}: ‘Therefore, we hypothesize that \emph{TP53} may be the key to resolving esophagogastric disorders.’ (Line 247)

(c) In subsection ‘Turning to non-small cell lung cancer’, we emphasize the new discovery of LUAD and LUSC: ‘Notably, \emph{PTPRD} is a novel candidate predicted by ModSDP within LUAD-specific gene set and may represent a promising target for future experimental validation.’ (Line 266) and ‘Therefore, we predict \emph{PTEN} and \emph{SGK1} might provide an effective therapeutic strategy for LUSC by mediating the FoxO signaling pathway.’ (Line 271)

(d) In subsection ‘Typical malignant tumors in males and females’, we emphasize the new discovery of BRCA and OV: ‘Furthermore, we identified novel predictions in these contexts, including \emph{MED12} and \emph{RABEP1} in BRCA, and \emph{MED12} in OV-specific candidate for further functional validation.

(Line 296)

(e) In subsection ‘Carcinogenic signaling in adults versus children’, we emphasize the new discovery of AML: ‘All remaining genes except for \emph{TNC} have been previously implicated in AML \cite{jalnapurkar2024phf6, hurtado2021asxl1, nepstad2020pi3k}. Thus we predict \emph{TNC} as a novel candidate by ModSDP.’

(Line 344)

(f) In subsection ‘Risk factors differ between smokers and nonsmokers’, we emphasize the new discovery of LUAD nonsmokers: ‘In addition, we presume that the mutated \emph{CYSLTR2} may be involved in lung cancer development through the production of aberrant GPCRs, based on the evidence observed in other cancer \cite{ceraudo2021direct}.’(Line 466) as well as HNSC smokers: ‘We predicted the T-cell leukemia virus-1 (HTLV-1) infection pathway as a novel finding in HNSC because we identified \emph{XPO1} and \emph{EP300} as smoker-specific biomarkers involved in this pathway.’ (Line 477)

(g) To more clearly highlight our new findings, we replaced ambiguous expressions with specific keywords. For example, we explicitly use phrases such as ‘...are novel predictions by EntCDP’ (Line 236), ‘...is a novel candidate predicted by ModSDP’ (Line 267), ‘We predicted ... pathway as a novel finding’ (Line 477), and ‘we hypothesize that... ’ (Line 247) to strengthen the presentation of our contributions.

(2) We added summary sentences at the beginning or end of major subsections to help catch key insights and the most important findings. For example:

(a) In subsection ‘Typical malignant tumors in males and females ’, we first highlighted the specific pathways between BRCA and OV as the most important results at the beginning of the paragraph: ‘For the differences, BRCA-specific analysis highlighted the T cell receptor (TCR) signaling pathway, whereas OV is specifically characterized by TNFR1 pathway activation.’ (Line 288)

(b) In subsection “Comparison of solid and liquid tumors”, we added a summary paragraph to hightlight the age-speific pathways within AML and GBM : ‘In summary, the PI3K-Akt signaling pathway (\emph{PIK3CA} and \emph{PTEN}) is critical in adult GBM, while PAK signaling (\emph{PTPRD} and \emph{PDGFRB}) is specific to pediatric GBM. ... Other genes, such as \emph{TNC} in adult AML and \emph{CEBPA} and \emph{ID3} in pediatric GBM serve as our predicted therapeutic molecular targets within their associated signaling pathways.’ (Line 369)

(c) In subsection “Biomarkers for subtypes of pediatric hematological and neural tumors”, we added a summary paragraph to hightlight subtype-specific pathways in neural tumors: ‘Overall, by comparing with PA, we identified subtype-specific pathways in neural tumors: PAK signaling (\emph{PTPRD} and \emph{PDGFRB}) in GBM, CFTR activity (\emph{PPP2R1A}, \emph{KLHL36}) in MBL, ...,while \emph{LRP1B} and \emph{PTPN11} appear to be NBL-specific candidates. These predicted genes are promising for follow-up studies on roles in signaling pathways.’(Line 430)

(d) We also added summarizing statements to clarify the purpose of doing the following work and the significance of these findings.

For the former, we add ‘The biological relevance of EntCDP is supported by the identification of the common gene set \{\emph{CTNNB1}, \emph{KRAS}, \emph{MET}, \emph{PTPN6}, \emph{TP53}\} for cohorts LUAD and LUSC’ before analyzing the commonalities between LUAD and LUSC (Line 254). Similar explanatory sentences were also added in Lines 322, 330, and 355.

For the latter, we added ‘This highlights the need to explore virus-related mechanisms in non-alcohol-associated tumor development’ after we searched for the virus-related signaling pathways (Line 489). Similar explanatory sentences were also added in Lines 406, 415 and 460.

(3) In Discussion section, we summarized our most important findings in each section and proposed several testable hypotheses. Specifically, we add sentences: ‘For example, EntCDP identified \emph{TP53} as an important gene implicated in gastrointestinal disorders. When looking at women cancers, disturbing ErbB signaling pathway may contribute to breast and ovarian cancer. ModSDP identified mTOR signaling pathway in Lung squamous cell carcinoma and FoxO signaling in lung adenocarcinoma, which can help with subtype heterogeneity understanding.’ in Line 564 and ‘Region-specific analysis reveals that PI3K-Akt signaling pathway regulated by \emph{HLA-A} and \emph{HLA-B} is involved in Chinese patients with bladder cancer compared to Americans,..., Besides, we hypothesized that NOTCH signaling mediates alcohol-related carcinogenesis, while obesity may promote cancer via CDKN-regulated pathways.’ in Line 571.

(4) We reorganized the structure of relevant paragraphs to highlight the most important and novel findings more clearly. For example:

(a) In subsection ‘Typical malignant tumors in males and females’, we first highlighted the specific pathways between BRCA and OV as the most important results, rather than examining the two types of cancer separately. (Line 288)

(b) In subsection ‘Biomarkers for subtypes of pediatric hematological and neural tumors’, we have reorganized the description of the four types of neural tumors to improve logical progression and thematic clarity:

·We first highlighted the commonalities between GBM and MBL, focusing on their common genes and overlapping targets identified when compared to NBL cohorts (Line 403). We also focused on the molecular differences between GBM and MBL; (Line 408)

·Then we analyzed the similarities between NBL and PA, and further noting the greater heterogeneity observed in NBL; (Line 417)

·Finally, by examining specific features of GBM, MBL, and NBL compared to PA, we summarized subtype-specific therapeutic signaling pathways and newly predicted genes of potential relevance. (Line 430)

(5) We deleted some less important paragraphs and put them in the supplementary materials.

In section ‘Targeting novel driver genes in region-specific signaling pathways’, we considerd that the discussion on cohorts lacking IntOGen genes is less important, so we remove the paragraph ‘Some cohorts of certain types of cancer lack information about driver genes provided by IntOGen, ..., in the tumor development of glioblastoma.’ from the main text to the supplementary materials (Section B in S1 Text). We then added a sentence in Line 146 to cite this part: ‘Since some cohorts lack IntOGen genes, we restricted hypergeometric test to cohorts containing IntOGen genes (Section B in \nameref{S1 Text}).’(Line 146)

(6) We also refined the language and sentence constructions to improve readability and ensure a clearer logical progression.

(a) We revised the structure by first demonstrating the identification results obtained through the EntCDP and ModSDP models, followed by a detailed analysis of these results. This approach ensures that the methodology and major outcomes are clearly stated before specific interpretations.

For example, in section ‘Turning to non-small cell lung cancer’, we replaced the sentence ‘\emph{KRAS} is one of the IntOGen genes of cohort LUAD and its mutation is a critical criterion in targeted therapy for patients diagnosed with LUSC \cite{ito2019targeting}. One pathway called the proteoglycans in cancer was discovered via the common gene set \{\emph{CTNNB1}, \emph{KRAS}, \emph{MET}, \emph{PTPN6}, \emph{TP53}\} for the two cohorts (Fig \ref{fig3}C top, \ref{fig3}D). ’ by ‘The biological relevance of EntCDP is supported by the identification of the common gene set \{\emph{CTNNB1}, \emph{KRAS}, \emph{MET}, \emph{PTPN6}, \emph{TP53}\} for cohorts LUAD and LUSC (Fig. \ref{fig3}C top, D). Among them, \emph{KRAS} ... Additionally, this gene set is enriched in the proteoglycans in cancer pathway. ’(Line 254)

(b) To improve clarity and better reflect the analytical process, we revised sentences that vaguely described the model’s results. For example, we replaced ‘Among the common genes of cohort ESCA and STAD identified by EntCDP’ by ‘By applying EntCDP to cohorts ESCA and STAD, we identified a common driver gene set when $K = 9$.’ (Line 231) Similar sentences were also revised in Lines 173, 240 and 507.

We hope these changes better convey the significance of our findings while addressing the reviewer’s concerns about experimental testability.

*3. The current figures are hard to follow by readers without reading the legends. The authors should replot the figures and add more labels to improve the readability.*

**Response:**

We appreciate the valuable comments of the reviewer. We have improved the readability of all figures by:

(1) Label clarity: adding clear legends and labels to axes and key elements.

Fig. 1B: Add column names to the box.

Fig. 1C: Add descriptions of solid and dotted lines and legends for “Y” and “N”.

Fig. 2A: Add a color legend to bar clarify the representation of each color.

Fig. 2B: Move the last proportion column forward to correspond to the fill bar, and label the proportion column.

Fig. 2C, D: Mark the key result boxes with dots corresponding to our models.

Fig. 3A,C,E, Fig. S2A: Add the number of genes, i.e. “K” to each box.

Fig. 3B,D,F: Add a color legend to genes in the pathway map to distinguish between enriched and non-enriched genes.

Fig. 4A: Add comment boxes with different colors for each box of result, to explain the comparative objects and the use of models.

Fig. 4B: Add comments to the arrows.

Fig. 5, Fig. S3: Add lagends to the pie chart and genes in the pathway map to distinguish between enriched and non-enriched genes.

(2) Structural reorganization: reorganized locations of some columns and gene names

Fig. 2B: Move the last proportion column forward to correspond to the fill bar, since they can supplement each other.

Fig. 5: Move gene names in the bottom heatmap to the right heatmap (A, C) and gene names in the top heatmap to the left heatmap (B), which allows the gene-annotated heatmap to show specific gene sets of exposure (top) and control (bottom) groups.

(3) Additional enhancements

To clearly distinguish the different colors in the legend, we make color substitutions for some of the elements of Fig. 4A: change the color of the GBM from blue to purple.

To show the relationship between the subgraphs and increase the readability, we added two-way arrows to Fig. 4B and C as well as D and E.

The revised figures now convey key messages more intuitively.

************
*Reviewer #2 (Comments to the Author):*

*Zhou et al. apply their recently developed two models, EntCDP and ModSDP to analyze mutation data. The author provides a comprehensive framework for understanding cancer signaling heterogeneity across diverse populations and clinical contexts. This is a compelling area of research, and I recommend publication with the following revisions.*

**Response:**

We thank the Reviewer for the positive evaluation and valuable comments on our work.

*However, I have thought about a couple of concerns.

1. What’s the differences between Ar and Bt in ModSDP model? How to determine the number of driver genes K? Further elaboration on these points would enhance clarity and comprehensibility.*

**Response:**

We appreciate the reviewer’s valuable comments on these two points.

(1) We first answer the differences between Ar and Bt in ModSDP model.

In the ModSDP model, Ar and Bt typically represent two distinct cancer groups. The goal is to identify signaling pathways that are specific to Ar relative to Bt. For example, Ar and Bt may correspond to the risk group and the control group of the same cancer type, respectively. In this case, we aim to investigate which signaling pathways are specifically associated with risk factors such as smoking, alcohol consumption, and obesity, compared to normal conditions. Conversely, if the control group is set as Ar and the risk group as Bt, then we are exploring which gene mutations, distinct from the known risk factors, might disrupt signaling pathways and lead to cancer in individuals without those risk factors. By analyzing both perspectives, we can identify distinct therapeutic targets for the two populations, facilitating personalized intervention strategies.

In fact, the desired result benefit from the setting of the ModSDP model, where in the objective function, the Ar group is treated as the minuend and the Bt group as the subtrahend. Under a maximization framework, each component of the Ar group is expected to be significantly larger than that of the Bt group. As a result, the identified gene sets demonstrate both high coverage and mutual exclusivity across all cancer types in Ar, but not in Bt. In other words, the gene set obtained by ModSDP represents a signaling pathway specific to the Ar group rather than the Bt group.

(2) We then explain how to determine the number of driver genes *K*.

The identification of cancer driver gene modules was first systematically addressed by Vandin et al. (*Genome Res., 2012*), who proposed evaluating gene sets of size *K* = 2 to *K* = 10. The lower bound *K* = 2 captures the minimal case of mutually exclusive gene pairs, while the upper bound *K* = 10 the reliability of the identified results within the sample size typically provided by large-scale cancer sequencing projects.

Inspired by this framework, we applied EntCDP or ModSDP to identify candidate gene sets for each *K* in the range 2 to 10. To assess the significance of each sets, we performed permutation-based statistical test (Method section: Line 644). Pathway enrichment analysis was subsequently performed on the significant sets using the DAVID tool. (Method section: Line 660).

For each set enriched in significant pathways, we calculated the maximum proportion of genes mapped to any single pathway. The gene set with the highest ratio was selected as the final candidate. For example, as illustrated in the revised Fig. 2B, we identify region-specific signaling pathways across different countries or regions and display the enrichment ratios of the identified gene sets within the corresponding pathways. This proportion actually represents the maximum ratio observed.

In the Methods section of our revised manuscript, we have updated the section title from ‘Pathway Enrichment’ to ‘Pathway enrichment and gene set prioritization’ (Line 660) to better reflect the expanded content. In this revised section, we have added a detailed description of the procedure used to select the most relevant gene set (i.e. the selection of *K*) based on enrichment ratio, as explained below.

‘Subsequently, for each gene set enriched in significant pathways, we quantified the enrichment ratio as the maximum proportion of genes mapped to any enriched pathway. The gene set with the highest enrichment ratio was selected as the final candidate for downstream analysis.’

*2. Could the method be applicable for identifying driver pathways across different subtypes of one cancer? For example, Luminal A, Luminal B, Basal of breast cancer.*

**Response:**

We appreciate the reviewer’s insightful question.

Yes, both EntCDP and ModSDP models are applicable for identifying driver pathways across different subtypes of a single cancer type. For example, in our manuscript, we applied it to compare two major subtypes of lung cancer—lung adenocarcinoma and lung squamous cell carcinoma (Line 249: subsection ‘Turning to non-small cell lung cancer’). We add the advantage of ModSDP in identifying subtype-specific pathways at the end of this subsection (Line 272): ‘These findings demonstrate the ability of ModSDP to recover biologically meaningful and therapeutically relevant gene sets specific to cancer subtypes.’

As long as subtype annotations are available, we can perform pan-subtype analyses to identify common pathways and contrastive analyses to highlight subtype-specific driver pathways. Specifically, for breast cancer, we can examine common pathways across Luminal A, Luminal B, and Basal-like subtypes, or investigate specific pathways in any one (or two) subtype(s) compared to the others (e.g., Luminal A vs. Luminal B, or Basal vs. Luminal A and B). Therefore, our method is flexible and can be adapted to compare any combination of subtypes based on the research priorities.
