# Supplementary material for: Multi-context modeling of driver pathways reveals common and specific mechanisms across 23 cancer types
Source: PLoS Comput Biol. 2025 Aug 6;21(8):e1013349. doi: 10.1371/journal.pcbi.1013349 (PMC12349879; doi:10.1371/journal.pcbi.1013349)
Supplement: Attachment — Submitted filename: Response_to_Reviewers_auresp_2.docx [file pcbi.1013349.s010.docx]

**Responses to the Reviewers’ comments:**

We would like to sincerely thank the editor and reviewers for their efforts in evaluating our manuscript. In this revised version, we have carefully addressed the editor’s comments regarding the language and grammar in our manuscript to improve its clarity and readability. We believe that these changes have further enhanced the manuscript. We would like to express our sincere gratitude to both reviewers for their positive evaluations and constructive comments, which have greatly improved our manuscript. The editor and reviewer comments are numbered, and presented below in blue italics. Our corresponding responses are provided in standard font.

*Editor (Comments to the Author):*

*1. Please note that at this stage, the reviewers have responded that you have addressed all of their scientific concerns. However I note as an editor that this manuscript still suffers from a number of grammatical and language issues that I would like to see resolved before acceptance of a final form of the paper. Please work with an English language assistance at your institution to reread and make final edits before we move to full acceptance of the paper and type-setting stage.*

**Response:**

We would like to thank for your careful consideration of our manuscript. We appreciate your feedback regarding the language quality. In response, we have thoroughly revised the manuscript to improve the grammar and overall language clarity. We believe that these changes have significantly enhanced the readability of our work.

Specifically, we have corrected spelling and grammatical errors, improved the use of tenses and prepositions, enhanced sentence structure and logical flow, and refined academic expressions to ensure the language meets the standards of PLOS Computational Biology.

All modifications have been clearly marked in the “Revised Manuscript with Track Changes” file, which has been uploaded along with the clean version of our revised manuscript.

Please find attached:

A revised version of the manuscript with track changes.

A clean version of the revised manuscript without tracked changes.

We hope that the revised manuscript now meets the journal’s publication standards and we look forward to your favorable response.

*2. If you would like to make changes to your financial disclosure, competing interests statement, or data availability statement, please make these updates within the submission form at the time of resubmission. Guidelines for resubmitting your figure files are available below the reviewer comments at the end of this letter.*

**Response:**

Thank you again for your consideration. Please find below our detailed responses regarding the financial disclosure, data availability, and figure resubmission:

(1) Financial Disclosure and Competing Interests Statement:

We have not made any changes to the financial disclosure or competing interests statement, as there are no updates required in these sections.

(2) Data Availability Statement:

We have maintained our previous data availability statement. Regarding Reviewer #2’s response of “None” to the data availability question, we would like to clarify that all data used in our study are sourced from public databases and are freely available for download and use. In our GitHub repository, we have provided data necessary for running the code, and all data can be accessed directly from the original public sources.

(3) Figure Resubmission:

As requested, we have re-uploaded all main text figure files using the PACE digital diagnostic tool to ensure they meet PLOS requirements. All figures in the main text have been processed and replaced with the PACE-validated versions.

*3. Journal Requirements:*

*1) We have noticed that you have a list of Supporting Information legends in your manuscript for Supplementary Figures (1-3). However, there are no corresponding files uploaded to the submission. Please upload them as separate files with the item type 'Supporting Information'.*

*2) We noted that the main figures are included in the "Files for compiling Revised_Manuscript.zip" with the Item Family "Supplemental." Please note that the main figures should be uploaded as separate figure files in .tif or .eps format with the Item Family "Figure."*

**Response:**

Thank you for bringing this to our attention. We have now uploaded the corresponding files for FigS1–3 as separate files in the required .tif format with the item type “Supporting Information” .

We apologize for the oversight regarding the submission of main figures. We have now uploaded all main figures as separate files in the required .tif format, each processed and validated using the PACE digital diagnostic tool, and assigned them the item family “Figure” as instructed.

************
*Reviewer #1 (Comments to the Author):

The authors have revised the manuscript according to the reviewers' comments and the manuscript is acceptable now.*

**Response:**

We sincerely thank Reviewer #1 for your positive evaluation and for acknowledging our revisions. We appreciate your constructive feedback throughout the review process, which has greatly improved our manuscript.

************
*Reviewer #2 (Comments to the Author):*

*The revisions made are satisfactory.*

**Response:**

We would like to thank Reviewer #2 for your encouraging comments and for recognizing the improvements made in our revised manuscript. We are grateful for your valuable suggestions during the review process.
